# Psychosocial Interventions for Amphetamine Type Stimulant Use Disorder: An Overview of Systematic Reviews

**DOI:** 10.3389/fpsyt.2021.512076

**Published:** 2021-06-17

**Authors:** Mai Thi Ngoc Tran, Quang Hung Luong, Giang Le Minh, Michael P. Dunne, Philip Baker

**Affiliations:** ^1^Faculty of Health, School of Public Health and Social Work, Queensland University of Technology (QUT), Brisbane, QLD, Australia; ^2^Center for Training and Research on Substance Abuse-HIV, Hanoi Medical University, Hanoi, Vietnam; ^3^Nursing and Midwifery Faculty, Hanoi Medical University, Hanoi, Vietnam; ^4^Faculty of Law, Australian Centre of Health Law Research, Queensland University of Technology (QUT), Brisbane, QLD, Australia; ^5^Institute for Community Health Research, Hue University of Medicine and Pharmacy, Hue University, Hue, Vietnam

**Keywords:** psychosocial intervention, cognitive behavioural therapy, contingency management, drug addiction, amphetamine type stimulants, synthetic drug, injection drug, meta-analysis

## Abstract

**Introduction:** Amphetamine-type stimulants (ATS) use is a global concern due to increased usage and the harm to physical, mental, and social well-being. The objective of this overview of systematic reviews is to summarise trial results of psychosocial interventions and describe their efficacy and safety.

**Methods:** We searched seven bibliographic databases to November 2020 for systematic reviews examining ATS misuse treatment by psychosocial interventions. Given the apparent incompleteness of the included reviews, we undertook a supplemental meta-analysis of all eligible primary studies.

**Results:** We included 11 systematic reviews of moderate to high quality and 39 primary studies which assessed the outcomes of psychosocial interventions on people who use ATS. The key findings include: (1) There were conflicting results about the effectiveness of psychosocial interventions among reviews, which may confuse decision-makers in selecting treatment. (2) In the supplemental meta-analysis, relative to usual care (only counselling or self-help materials), membership of a psychological intervention group was associated with an important reduction in drug usage [risk ratio (RR) 0.80, 95% CI: 0.75 to 0.85]. Patients in psychological interventions used injectables substantially less [odds ratio (OR) 0.35, 95% CI: 0.24 to 0.49]. The risk of unsafe sex in the psychosocial intervention group was lower than in the control group (RR 0.49, 95% CI: 0.34 to 0.71). The combination of therapies reduced 1.51 day using drugs in the preceding 30 days (95% CI: −2.36 to −0.67) compared to cognitive behavioural therapy intervention alone. (3) Compared to usual care, cognitive behavioural therapy was less likely to be retained at follow-up (RR 0.89, 95% CI: 0.82 to 0.97; high-quality evidence). However, the additional of contingency management strategy can make an important improvement upon retention (RR 1.42, 95%CI: 1.25 to 1.62).

**Authors' Conclusions:** Integrated models are more effective than a single-treatment strategy. Comprehensive and sustained psychosocial interventions can help to reduce use of ATS and other drugs, risk behaviours and mental disorders, and significantly improve treatment adherence.

## Introduction

### Description of the Condition

Amphetamine-type stimulants (ATS) are the second most common illegal drugs used globally after cannabis, and their usage has been spreading quickly in diverse populations (1, 2). ATS comprises two sub-groups: meth/amphetamine and ecstasy. UNODC estimated that 27 million people used amphetamines and 21 million people used ecstasy in 2018 (3). However, there is a notable point at which the global quantities of ATS seizures (based on kilogramme equivalents and population growth) were highest among drugs from 2010 to 2018 (4). Particularly, ATS precursors with new types of chemical designs were outside of international control (5). Therefore, the ATS market was considered complicated and uncontrollable. Although ATS are most used in Asia and North America, ATS are an attractive drug and associated with dynamic lifestyle of users. Further, the risks of ATS use are often underestimated (6). ATS are highly addictive and can cause harm to physical, mental, and social health (7, 8). The purity and chemical composition of ATS often are unclear (9, 10). Prolonged ATS misuse can cause mild to severe mental and physical disorders including malnutrition, aggression, sleep disorder, nervous stress, hallucinations, and psychosis (11).

### Description of the Interventions

A diverse array of psychosocial interventions have been applied to treat substance use disorders, particularly for amphetamine users (12). The broad aims of psychosocial therapies are to help drug users understand the harmful effects of ATS, recognise their personal values and strengths, and overcome social stigma and self-stigma to engage in specialised individual or group therapies. Knapp et al. (13) found people tend to seek alternative activities to replace drug use behaviours when they understand the withdrawal symptoms, risky social influences, and coping strategies to prevent drug use. Psychological therapy continues to be developed in parallel with understanding predictors of usage, the needs of users, and the changing social circumstances of ATS users. This review focuses on the most common psychosocial treatments for ATS that have been studied worldwide, as below.

*****Cognitive behavioural therapy***** (CBT), introduced by Bandura (14) and based on the principle of learning through an individual's experience and interaction, has been widely adapted to support ATS users in improving their coping skills, preventing relapse, widening service outreach, and promoting community reinforcement (15). Meanwhile, *****brief intervention therapy (BI)***** shows measurable outcomes in ATS users who have low levels of dependence when they receive concise and focused counselling from health care providers to stimulate positive changes and increase their coping competency (16). *****Contingency management (CM)*****, which encourages the use of rewards for satisfactory progress or behavioural changes (17), has also been utilised in ATS and other substances treatment when prizes such as money, vouchers or even clinic privileges are employed to reward for abstinence behaviour (18). *****Mindfulness*****, another therapy originated from the Buddhism and Hinduism religions (19) is considered a promising therapy to deal with ATS as it helps ATS users avoid unwanted thoughts of drug-related pleasure, craving or emotional injury (20). There have been several adapted interventions using mindfulness mediation to treat ATS use such as a combination of psychoeducation, coping strategies and breath meditation, loving-kindness meditation, and written reflection (21). *****Motivational interviewing***** therapy, which applies the stage of changes in counselling to encourage treatment linkage and retention (22), has also been adopted by health care providers to identify drug users' present stage to help them evaluate the pros and cons of their behaviours and resolve ambivalence (23).

*****Twelve steps facilitation***** is the approach of using a person with higher power to facilitate the giving-up process and prevent relapsing in ATS users using alternative activities to assist their acceptance, surrender and active involvement (24, 25). *****Casemanagement***** has been widely used in Western countries to assist ATS and drug users to connect with health and human services and get access to clinical support while empowering themselves and valuing the support of their family and community (26, 27). *****Therapeutic community or community reinforcement therapy***** has broadly been adapted to assist ATS and drug users by facilitating self-help groups (28), changing the surrounding environments with social incentives or providing stable houses (29). *****Family therapy***** has been modified with various models such as the family disease model, family system model and behavioural model, to assist drug users and their family to identify and strengthen each member's proactive roles in dealing with the problem (30–32).

Each of the psychosocial therapies mentioned above has their own advantages and has been proved effective somewhere in the world in term of ATS and drug abuse treatment. Currently, the matrix model, which integrates various psychosocial treatments and set the therapeutic plan for drug users each day during the treatment process, is predominantly accepted as the advanced therapy (33).

### Why Is It Important to Do This Overview?

Numerous studies have described the outcomes of psychosocial interventions to reduce illicit drug use. In the past two decades, there has been significant growth in the number of intervention studies using psychosocial therapies to minimise harms from ATS use, and subsequently, many systematic reviews have been published. The increasing number of reviews and individual studies can be overwhelming for policymakers and health program planners who need clear, practical guidance about ATS programs (34). There are several gaps in the literature about the efficacy of psychosocial treatments to reduce harms related to ATS use. First, the increasing number of individual studies and systematic reviews has revealed inconsistency in evidence about effectiveness. For example, a recent review has shown that the quality of evidence in many primary studies was low and insufficient to show the efficacy of CBT in treating ATS misuse (35). This conflicts with conclusions of other reviews supporting the effectiveness of CBT and other psychosocial interventions (36, 37). There is considerable heterogeneity among primary studies in the quality of evidence and this crucial element has not been adequately considered in some systematic reviews. A further complication is that protocols for interventions are continuing to emerge (38) and are applied in ATS programs. The increasing number of studies, controversies around divergent results, and little systematic meta-synthesis produce a situation where it is difficult for clinicians, policymakers and program planners to discern what therapies and treatment environments are beneficial. Overviews of systematic reviews can be useful to summarise evidence of reviews of different interventions from various communities and clinical settings. This overview set out to examine the quality of evidence from both systematic reviews and moderate to high-quality primary studies. The purpose was to add clarity and provide practical guidance for future interventions.

## Methods

**Figure 1:**
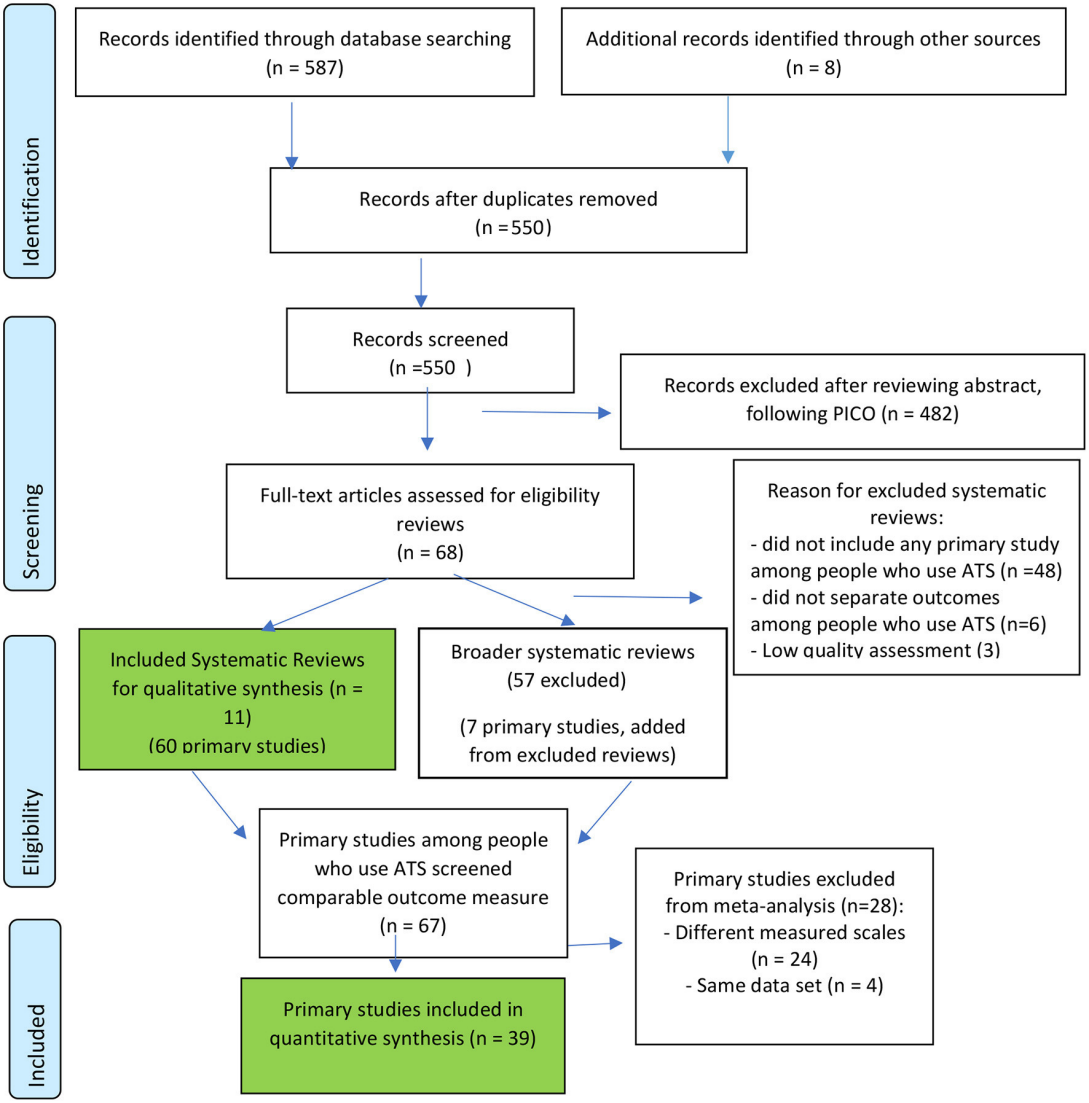
Review and study flow diagram.

Figure 1 shows the search and selection process for reviews and primary studies.

### Step 1: Search for Systematic Reviews

We included all published systematic reviews meeting the accepted definition of systematic reviews (39) evaluating interventions aimed at reducing ATS usage. We included eligible reviews summarising findings either narratively or statistically with a meta-analysis.

#### Systematic reviews were included in this current overview if they met the following inclusion criteria:

- *Study design:* Systematic reviews of randomised control trials (RCTs), quasi controlled trials and controlled cohort studies.
- *Types of participants:* Users of ATS, adults 18 years old, or above.
- *Types of interventions:* Any psychosocial interventions (psychosocial interventions, family/school/community-based interventions, and digital platform interventions/individual or multi-component interventions) designed to change ATS usage and harm reduction.
- *Comparison:* Usual care (self-help materials only or usual healthcare consultations), non-active treatment arm.
- *Types of outcome measures: containing any of the following outcomes*.

#### Primary outcomes:

- Change in ATS use (proportion of patients using ATS, abstinence, or relapse).
- Adverse events (unfavourable outcomes that occur during or as the result of the interventions).

#### Secondary outcomes:

- Change in related harm of drug use (HIV/AIDS risks, sharing syringe, unprotected risky sexual behaviours, physical and mental disorder, crime, and social issues).
- Follow-up at any time from treatment initiation.

#### Exclusion criteria:

Systematic reviews that did not include any primary studies among people who use ATS or did not specifically analyse the effects of psychosocial intervention, for example, measure cost-effectiveness.

#### Search Methods for Identification of Reviews

We searched four specialist databases of the Cochrane Database of Systematic Reviews (CDSR), Centre for Reviews and Dissemination (CRD), Health Evidence Registry of Reviews (http://healthevidence.org), and PROSPERO prospective registry of reviews. Furthermore, we searched three standard bibliographic databases: Medline (via separately Ebscohost and Pubmed), Scopus, and Web of Science. Additionally, the reference lists of included systematic reviews were scanned to identify additional eligible documents. A citation search was made of those papers included in the reviews. Several authors of relevant papers were contacted regarding any further published or unpublished works.

Search terms for all databases are presented in Supplementary Table 7. The retrieved data was download in the unit of 200 publications by using the “output records” and “save as text format.” The final dataset was transferred to Endnote for further analysis.

### Step 2: Search for Evidence From Included Primary Studies

From 68 potentially eligible systematic reviews, we identified 332 relevant included primary studies and continued to review abstracts. We excluded studies that targeted participants who were not people who use ATS, for example, non-specified drug users, other types of stimulant users. Finally, from 11 included reviews, we selected 60 primary studies among people who use ATS for full-text review. An additional seven eligible primary studies which were absent from the included reviews were identified from excluded reviews. Primary studies containing a standard primary or secondary outcome measure were selected for meta-analysis (39 studies).

Searching and data extraction was undertaken independently by two authors (MT and HL) based on the standardised electronic data extraction form. A third reviewer (PB) checked all the extracted data for accuracy and consistency. Data were managed by Endnote X9 and Rev Man 5.4.

### Step 3: Data Analysis

#### Quality Assessment of Systematic Reviews and Included Primary Studies

Systematic reviews: The assessments were undertaken by two independent assessors (MT and HL) with the Health Evidence Quality Assessment Tool (HE-QAT) (40). This validated tool contains 10-questions, yielding a maximum score of 10. Reviews are classed according to 8–10 “strong,” 5–7 “moderate,” and a score of 4 or below “weak.” All low-quality reviews (HE-QAT score < 5) we excluded. We resolved any disagreements by consensus among all review authors.

Included primary studies: Risk of bias of primary studies were assessed by Risk of Bias Tool following the Cochrane collaboration guidelines (41). The primary studies assessment was then compared with those described in the systematic reviews.

#### Data Analysis

##### For Narrative Analysis

The contents of 11 systematic reviews were extracted to consider the similarities and differences among them. Supplementary Table 1, extraction includes the review objectives, publication sources and the search dates, characteristics of included studies, population characteristics, intervention characteristics, and comparison of interventions and outcomes.

##### For the Meta-Analysis, Individual Studies Were Re-analysed

– Grouped according to intervention type. Examples of the categories include: contingency management; cognitive behaviour therapy.

– Sorted further by the control group, the scale of measurement, and timing of measurement: Each outcome from all primary studies were assessed whether they contained a standard outcome measure suitable for meta-analysis; reducing the number of primary studies to 39. Extracted data were analysed with RevMan 5.4.1. The selected outcomes included:

- **% Drug use at the end of treatment**: measured by the number of people who use drugs in each group (control and intervention); by a urine test or hair test; after completing treatment. Then we categorised this outcome into 3 different groups following type of intervention: contingency management, cognitive behaviour therapy and combination of different psychosocial therapies. The comparison is treatment as usual or no treatment.
- **The number of days using drugs in the past 30 days**: measured by mean; standard deviation by self-report; after completing treatment. Then we categorised it into two groups: combined psycho-social therapies compared to CBT only and CBT compared with no treatment.
- **% Follow up treatment until the end of treatment**: measured by the number of people who attend every intervention activity; by supervision report, right after completing treatment. This outcome was categorised into two groups: contingency management compared to non-contingency management; CBT compared with treatment as usual.
- **% Report unsafe sex at the end of treatment:** measured by the number of people who self-report about unsafe sex activities such as condomless anal intercourse; unsafe sex because methamphetamine use; by self-report of participants; at the end of treatment. There is one group: CBT treatment compared to the inactive treatment.
- **% drug injection at the end of psychosocial treatment compared to control:** measured by the number of people who self-report about drug injection.
- **Beck Depression Score at the end of psychosocial treatment compared to control**: measured by mean and standard deviation after completing treatment by BDI scale.

– Finally, the quality of evidence was GRADE assessed using the GRADE Pro GDT website, following Cochrane guidance (42). The GRADE table presented the summarised findings of internal and external validity of evidence and effect size. The certainty of evidence was GRADE assessed as in Supplementary Table 6.

## Results

### Description of the Included Systematic Reviews and Primary Studies

The included reviews had been published between 2008 and 2020. Within the 11 included systematic reviews, eight reviews focused on people who use ATS and three reviews were broader including ATS and other types of drug use such as cocaine and poly-drug users. The detail characteristics of reviews are presented in Supplementary Table 1.

#### The Methodological Quality of the Included Reviews

Of the 11 included reviews, 6 were of strong methodological quality (scores 8–10, low risk of bias); 5 reviews were of moderate quality (scored 5–7). The reason for scoring is explained in Supplementary Table 1 (Characteristics of included systematic reviews). The quality of evidence is presented in Supplementary Table 2 (Quality of reviews). We found the number of systematic reviews has increased each year on this topic, while the quality of research remained unimproved. This overview identified redundancy, in that many primary studies were included in more than one systematic review. Several systematic reviews had the same objectives and primary studies but were published in different journals. The common limitations of systematic reviews included lacking a comprehensive search. Reviews often combined differing types of interventions and differing populations. Further, often the included studies were not assessed for risk of bias (method quality is low). The review process often lacked transparency of how the two reviewers operated. Heterogeneity was apparent in reviews but lacked adequate explanation.

Among the 11 systematic reviews, ten reviews identified psycho-social interventions as effective in reducing drug usage, risk behaviours and mental disorders. However, only three reviews included a meta-analysis as the analytical method, and just two reviews evaluated the quality of evidence through GRADE assessment. In these two reviews, the reliability of evidence was graded low or moderate because of heterogeneity in the measured outcomes, and imprecision from small sample sizes. It should be noted that most primary studies took place in developed countries where the standards of care and treatment were relatively high.

#### Quality of Included Primary Studies in the Supplemental Meta-Analysis

**Figure 2:**
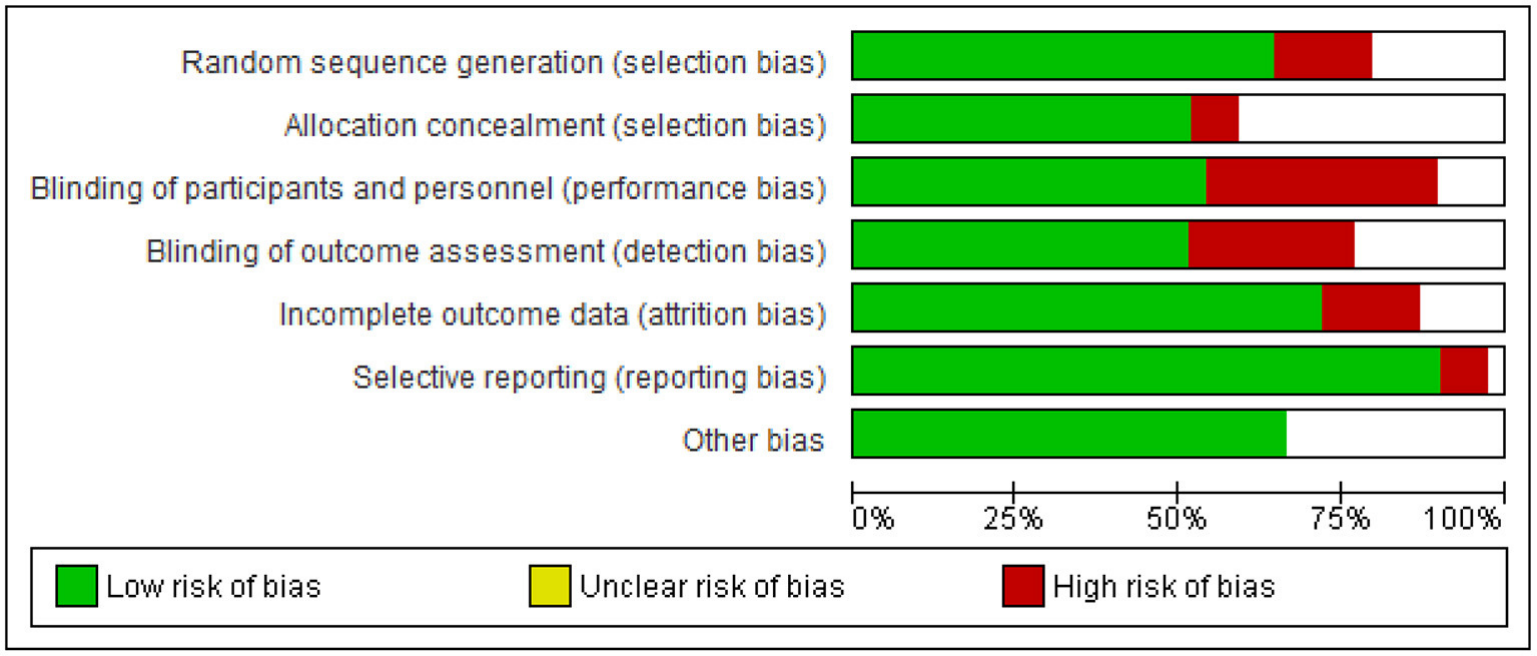
Methodological quality graph: review authors' judgements about each methodological quality item presented as percentages across all included studies.

The 39 primary studies included in the meta-analysis were assessed for risk of bias (Figure 2):

The detailed risk of biases of each included primary study is described in Supplementary Table 3. Inadequate blinding was most common, resulting in performance and detection bias.

#### The Heterogeneity of Primary Studies Among People Who Use ATS

Reviews typically identified the heterogeneity of primary studies and they did not summarise the differences among primary studies, yet they grouped them together. This overview summarised all outcomes measured from systematic reviews and primary studies (Supplementary Table 4). We found often outcomes were inconsistently measured by a plethora of tools, and outcomes measured by the same tool were sometimes reported inconsistently. Additionally, in 67 primary studies among people who use ATS, there was heterogeneity in the intervention type (Supplementary Table 5).

In this overview, for meta-analysis, we focused on the three most common psychosocial interventions. Among these interventions, CBT, CM, and combined different therapies were the most popular approaches, by both review and study numbers.

### Effect of Interventions

#### Effects of Interventions on Drug Use

Systematic reviews reached mixed conclusions about the effect of psychosocial interventions in decreasing drug use. Further, there are differences in evaluation about the quality of evidence. Ten of 11 systematic reviews demonstrated that psychosocial intervention could reduce the frequency, dose and risky routes such as injection of drug use. However, Harada et al. (35) concluded that the overall quality of evidence was low and insufficient to determine the effectiveness or ineffectiveness of CBT. Additionally, systematic reviews showed the benefit of combining different therapies to lengthen the duration of drug abstinence, compared with single therapy (13, 15). For example, combining CBT with CM provided a greater sustained reduction in drug usage than CBT alone (15). However, the quality of evidence was graded low (37, 43). Two conflicting conclusions about the effectiveness of the psychosocial intervention on reducing drug use and the high effectiveness of combined therapies were re-evaluated in this overview.

**Figure 3:**
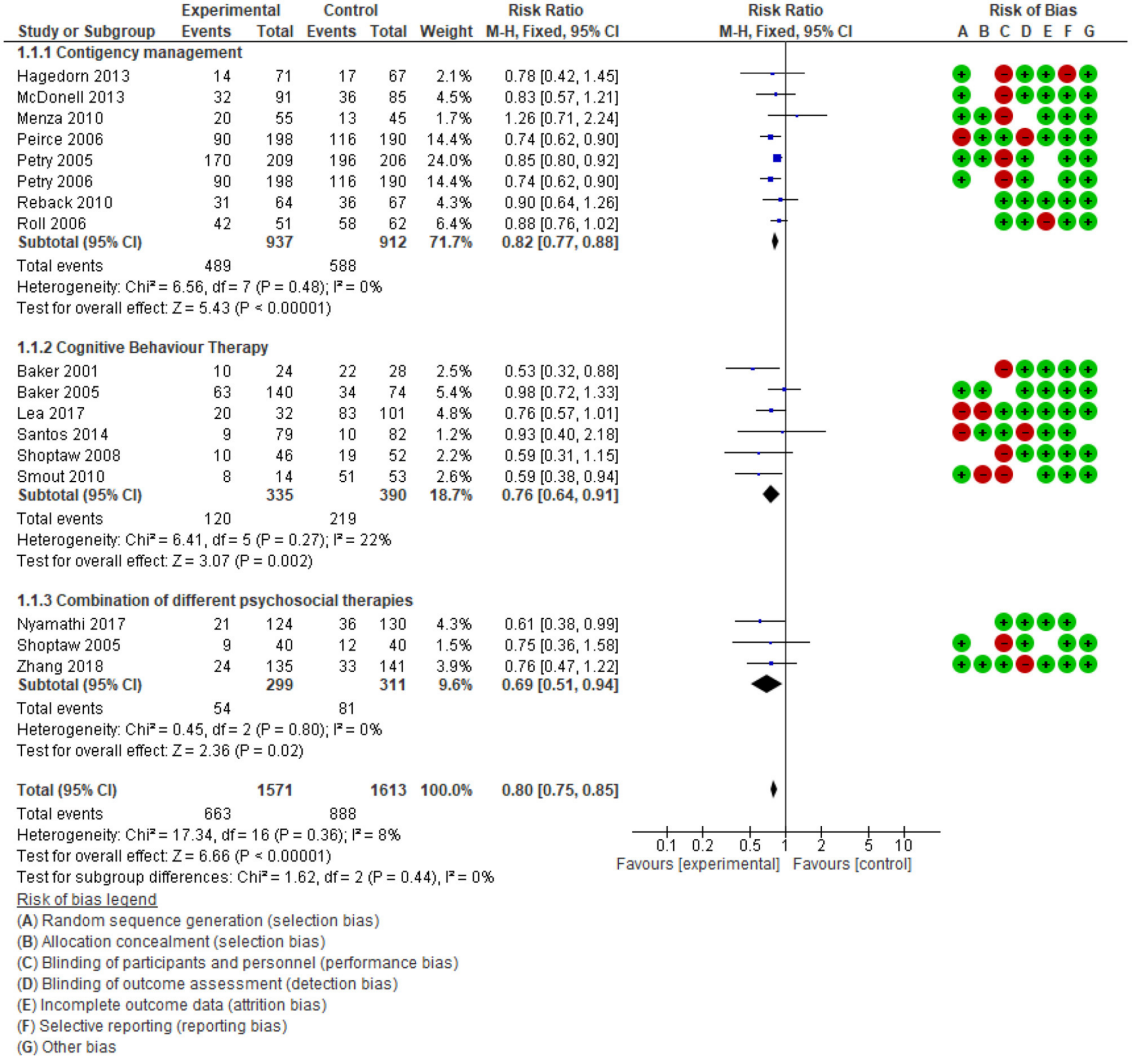
Comparison % Drug use at the end of treatment.

**Figure 4:**
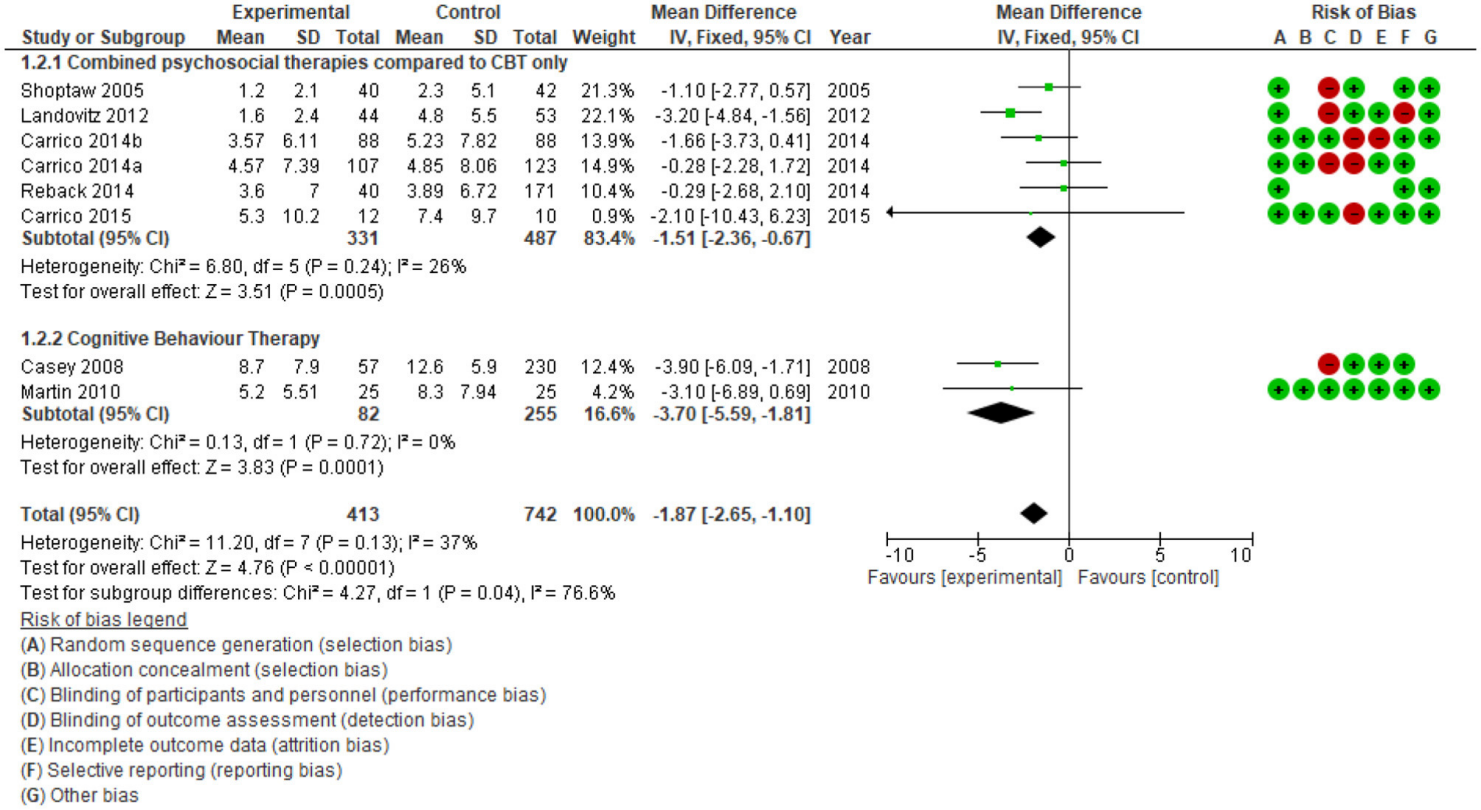
Comparison number of days using drugs after treatment, last 30 days.

In this overview, a supplemental meta-analysis (a re-analysis of studies) and GRADE assessment of evidence were conducted to examine the effect size of psychosocial intervention and the importance of evidence. First, the effect sizes on reducing drug use were estimated by two analysis: 1. comparison of the drug use percentage at the end of treatment between psychosocial interventions and treatment as usual (Figure 3) and 2. comparison number of days using drugs at the end of treatment during the last 30 days (Figure 4). Secondly, the quality of evidence was re-evaluated in Supplementary Table 6.

To assess drug use status following interventions, primary studies reported results of device-measured urinalysis (21, 44, 45) or self-report (46, 47) with variety of tools. For standardised reporting, we describe drug use outcome measured by a biological test (urine, hair) (Figure 3). Additionally, the effect of psychosocial interventions on reducing drug use was categorised into three groups (CM; CBT; any “combination” of differing psychosocial therapies). By categorising interventions and using a single type of outcome before aggregation, the results showed a high homogeneity of included primary studies (I^2^ = 0). The effect on reducing drug use was confirmed in all three groups of interventions. The relative risk of CM, CBT and combined different therapies are (RR) 0.82; 0.76; 0.69, respectively. Overall, 18 studies among 3,184 participants showed significant drug use reduction among people who were in the intervention group (RR 0.80, 95%CI: 0.75 to 0.85), the quality of evidence is high.

To ascertain consistency in the effect of combining different therapies, meta-analyses were performed with two objectives: 1. compare the impact of multiple therapies with CBT only (CBT); and 2. compare CBT alone with no intervention (Figure 4). Meta-analysis showed the average number of days using drugs in the preceding 30 days by people exposed to combined multiple psychosocial therapies is lower 1.51 days (95% CI: −2.36 to −0.67) than those in the CBT group alone, *n* = 868, studies = 7. Additionally, CBT reduced drug use by 3.7 days (95%CI: −5.59; −1.81 to *n* = 337, *n* = 337, studies = 2) compared to control groups with no intervention. The heterogeneity in each comparison is low (I^2^ < 30%), so that the effect of multiple psychosocial therapies in reducing the frequency of drug use and lengthening the time of drug abstinence after treatment is robust.

Review identified psychosocial interventions could reduce the frequency of use but not produce abstinence completely. There is limited evidence about the long-term benefits of psychosocial interventions. In Figure 3, the probability of drug use in the intervention group at the end of the treatment periods is 42.2% (663/1,571) while in the control group, 55.1% (888/1,613). However, as shown in Figure 4, the number of days using ATS reduced significantly compared to the control group. The included systematic reviews also indicated that the effectiveness subsequently declined. Stuart et al. (48) concluded that higher numbers of intervention sessions were associated with greater improvement. The intervention effect was often evaluated at the end of treatment or 3, 6, or 12 months after treatment. The effect appears highest at the end of treatment, then diminishes over time (15, 23, 37). This observation suggests psychosocial intervention may need to be repeated. Unfortunately, none of the studies compared the effectiveness of different timing and repetition of the intervention on the same group of participants. Moreover, the 11 systematic reviews also did not identify the optimal duration of a repeated intervention.

#### Effect of Psychosocial Interventions on Retention in Care

The relationship between psychosocial interventions and retention in treatment is unclear from the findings of systematic reviews. While Ciketic et al. (49) in a narrative analysis described the retention improvement among people who use ATS and treated by CBT or counselling, Minozzi et al. (37) showed the reduction in the dropout rate was not meaningfully improved. This result is re-evaluated by our overview. After comparing differences in intervention types amongst primary studies, they were categorised into two groups. Group 1 contained primary studies that included a CM intervention; group 2 contained primary studies of CBT, motivational interviews, and others (excluding CM).

**Figure 5:**
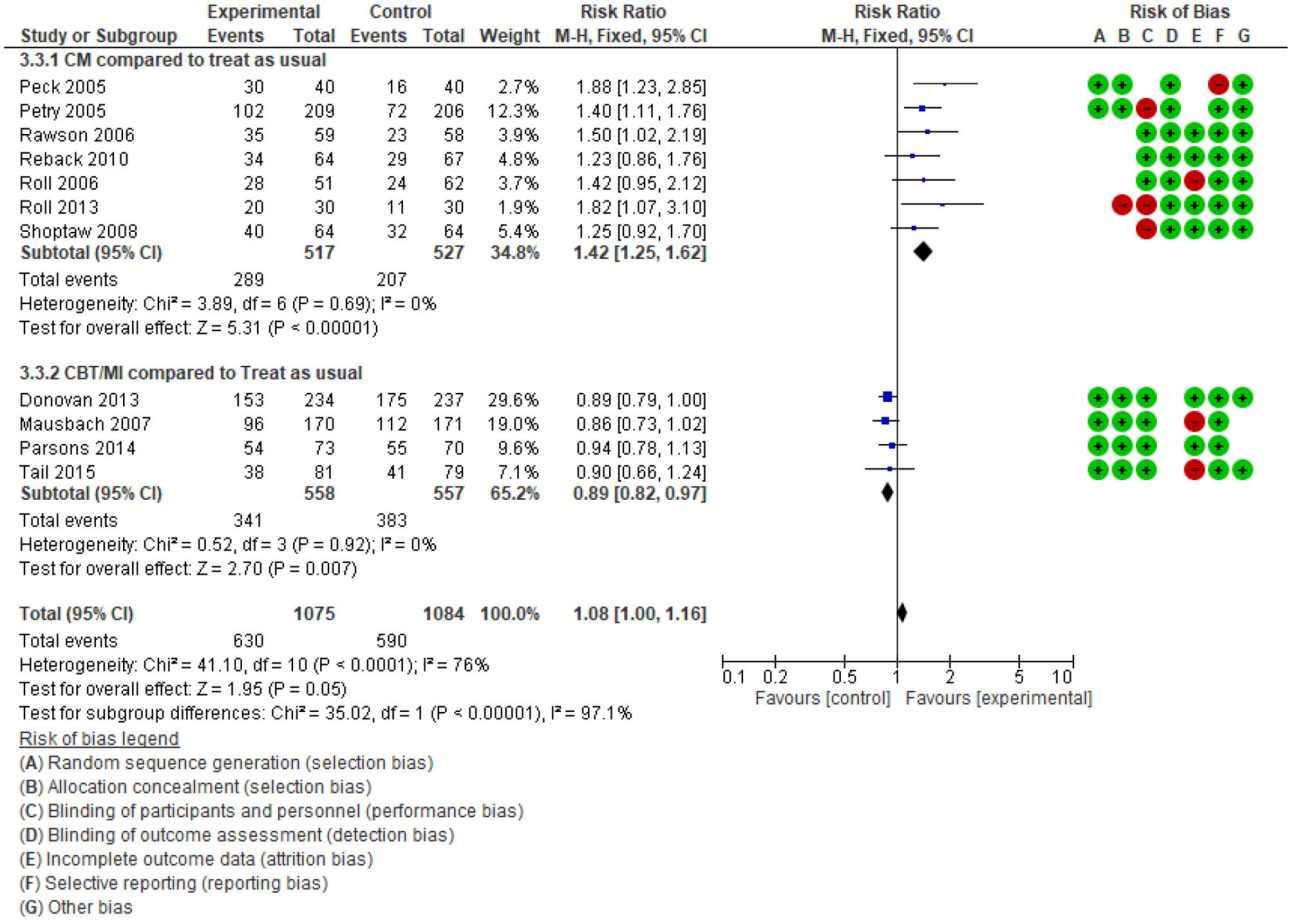
Comparison percentage of follow up treatment until the end.

The meta-analysis summarised in Figure 5 shows an important difference for both groups against the comparison. When psychological interventions were combined with CM, there was a large increase in the follow-up rate RR = 1.42 (95%CI: 1.25 to 1.62, *n* = 1,044, 7 studies, high-quality evidence). In contrast, for the interventions which were not in combination with CM, participant retention decreased (RR = 0.89; 95% CI: 0.82 to 0.97; *n* = 1,115; 4 studies, high quality evidence). In contrast to the individual reviews, this overview's supplemental meta-analysis differentiated between strategies intended to retain their study's participants.

This analysis identified that treatment program drop-out was a commonly reported problem among ATS users in psychosocial intervention; however, rewards such as coupons and money significantly improved the retention rate among drug users without severe mental disorders.

#### Effect of Intervention on Mental Disorders

**Figure 6:**
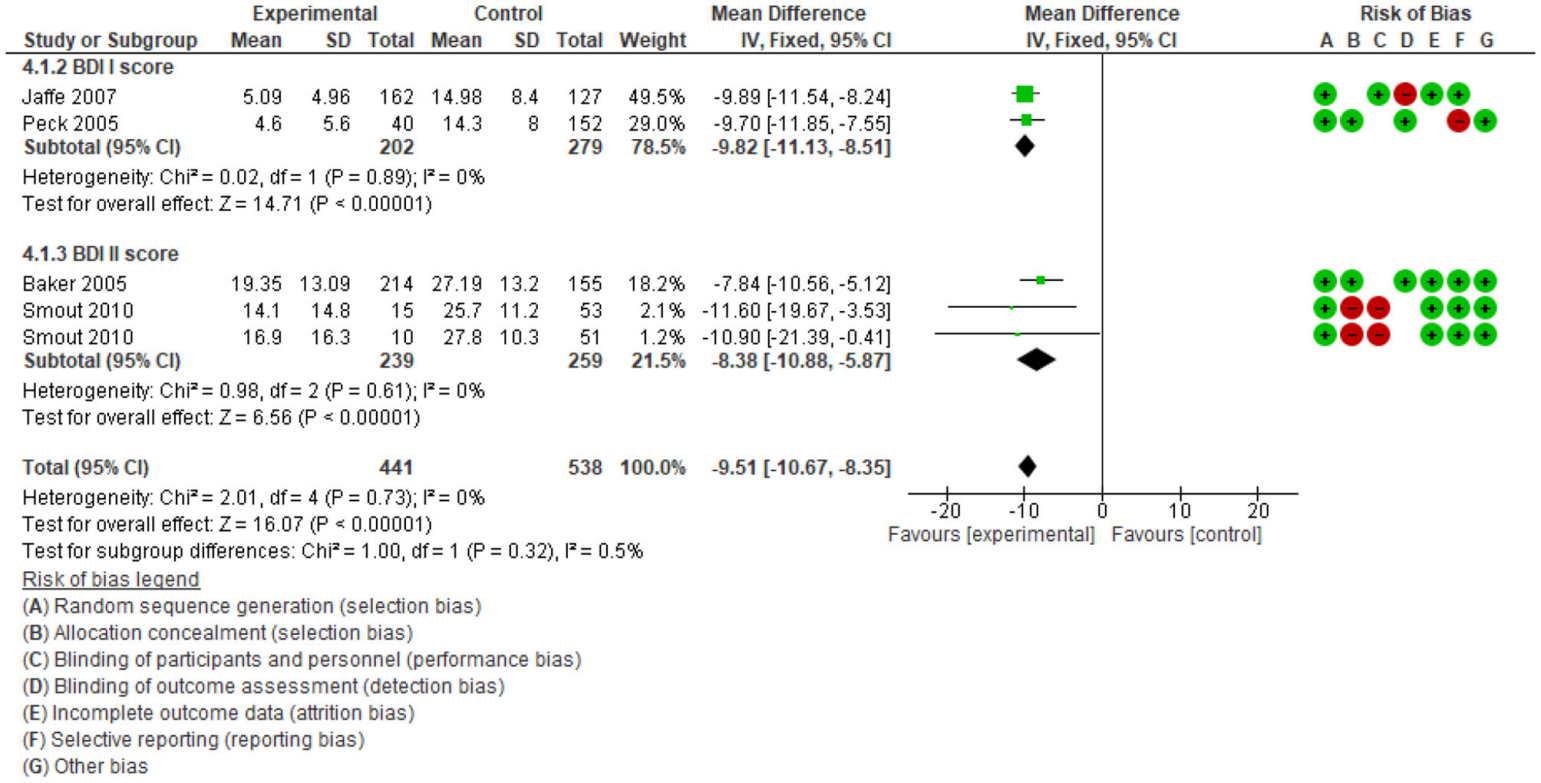
Comparison Beck Depression Inventory Score.

The overview of previous meta-analyses and our supplemental meta-analysis results differed in relation to the effect of psychological interventions upon psychiatric symptoms among people who use ATS. Four of the 11 systematic reviews evaluated the effect of psychosocial interventions on psychiatric symptoms. Psychiatric symptom outcomes included craving, the severity of dependence, depression (15, 37) and psychiatric distress (48, 50). While reviews with a narrative analysis identified positive impact on reducing craving, depression and anxiety, the review with a meta-analysis identified a non-significant difference between the intervention and control group (37). There is a lack of evidence in systematic reviews on the effect size of psychosocial interventions on psychiatric outcomes among people who use ATS. The heterogeneity in reporting outcomes (as shown in Supplementary Table 4) and small sample size were limitations for previous meta-analyses. This overview's supplemental meta-analysis indicates that psychosocial interventions decrease depression [when the Beck Depression Inventory (BDI) score was compared, see Figure 6].

The supplemental meta-analysis showed good certainty in the ability of psychological interventions to reduce depression. It should be considered that both versions of the BDI (I and II scales) each have 21 questions, with four possible responses, and scores ranging from 0 to 63 points. However, each scale differs in its application of cut-off points for these questions. As a result, studies that used BDI I or BDI II were initially analysed separately. Specifically, BDI I: 0–9 indicates minimal depression; 10–18 mild depression; 19–29: moderate depression; 30–63 severe depression. On the other hand, BDI II: 0–13: minimal depression; 14–19: mild depression; 20–28: moderate depression; 29–63: severe depression). Combined, the mean difference of the scores is −9.51 (95% CI: −10.67 to −8.35); *n* = 979, studies = 5, favouring psychological interventions. The heterogeneity is low (I^2^ = 0), showing good consistency.

Despite the increasing number of primary and systematic reviews, there remains a paucity of psychosocial intervention evidence on reducing psychiatric symptoms among people who use ATS. First, there is little published information on the frequency of specific psychiatric symptoms (craving, severe dependence, anxiety, manic, schizophrenia) in people who use ATS, and whether they can be reduced by psychosocial therapies. Second, there is a lack of evidence on how best to treat people who use ATS while having a severe mental disorder.

#### Interventions Targeted HIV/AIDS Risk Behaviours

The dual analysis of systematic reviews and their primary studies suggest psychosocial interventions can meaningfully reduce HIV/AIDS risk behaviours amongst drug users (36, 51). In particular, the review found high engagement in risk reduction programs for positive HIV patients who used ATS and received psychosocial therapies (36). Outcomes for sexual risk behaviours included reducing the number of sex partners (52) and increasing condom use (53). Decreasing drug use risk behaviours included reducing injected drugs (54) and reducing drug use during sex (55). However, no data were found on the association between psychosocial interventions and HIV/AIDS risk behaviours in the 11 included systematic reviews.

**Figure 7:**
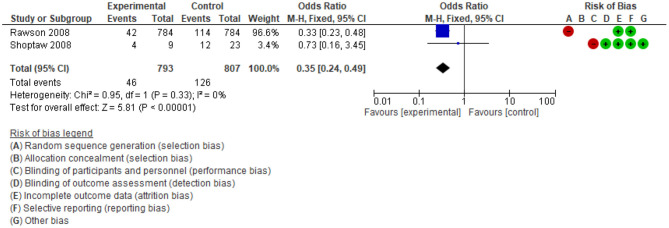
Comparison of percentage Injection drug at the end of treatment.

**Figure 8:**
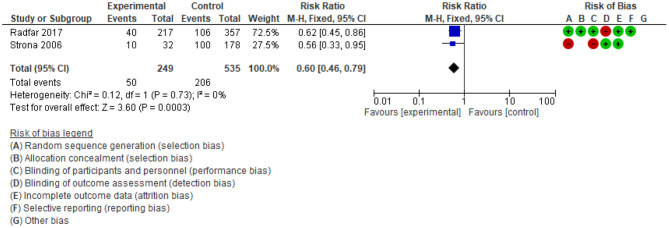
Comparison of unsafe sex risk behaviours by self-reported.

Two supplemental meta-analyses were conducted of four separate studies. In Figure 7, the odds ratio of injection drug reduction favoured the psychosocial intervention, compared to the control group (OR = 0.35; 95%CI: 0.24 to 0.49; *n* = 816; 2 studies; low-quality evidence). Analysis in Figure 8 showed that the risk of unsafe sexual behaviours among people in the psychosocial intervention group is probably lower than in the control group (RR: 0.6; 95%CI: 0.46 to 0.79; *n* = 784, 2 studies; moderate-quality evidence). Although these findings have low or moderate-quality evidence, there was a meaningful difference between the psychosocial intervention and control groups.

#### Adverse Effects

Interventions can generate unwanted effects, often referred to as adverse effects, side effects or adverse events (42). Only two included systematic reviews (37, 56) included statement of intent to analyse adverse events, however, no data were reported. Thus, consideration of adverse events was absent from the included studies.

## Discussion

### Summary of Main Findings

This overview contributes by improving the evidence-base on psychosocial treatment efficacy among people who use ATS. Previous systematic reviews identified low-quality evidence of reducing drug use (37), increasing mental health (57), reducing injection and sexual risk behaviour. Other reviews had significant heterogeneity and inconclusive outcomes (35). This overview synthesised evidence from several systematic reviews and included primary studies, identifying significant differences in outcomes between the primary studies in these 11 reviews. Our supplemental meta-analysis, a re-analysis of trial data, however, provides high-quality evidence for the most important outcomes. This overview of reviews identified the reasons for contradictory findings among the available systematic reviews. Additionally, compared to previous systematic reviews, the heterogeneity in the supplementary meta-analysis is much lower. This overview presents higher-quality evidence that psychosocial interventions probably reduce drug use, depression, and HIV risk behaviours. Supplementary Table 6, provides interpretative statements that summarise the overall effects and evidence quality.

### Interventions to Reduce ATS Use as Well as Other Types of Drug Use

This overview showed psychosocial interventions probably reduce the frequency of drug use among people who use ATS. The findings support recommendations for developing future comprehensive and short-time interventions. Combining psychosocial therapies appears essential to sustain the reduction of drug use and sustain participation. Comprehensive interventions such as matrix models show positive effects (58). The long-term effectiveness of individual therapy appears dependent upon social contexts and individual circumstances. Individual therapies appear to provide little benefit after 1 year of treatment (59). Combining differing effective therapies has the potential to reduce boredom and increase adherence (60). Therapy combination, applying short implementation intervals, with incentive rewards appear key to improving intervention quality. An implication for practise is that psychological therapies should be repeated to achieve outcomes (23).

### Interventions Targeted to Retention in Care and Mental Disorders

The included systematic reviews showed retention in care predicts drug use reduction and harm minimisation. To improve retention rates, it appears important to adopt different models of CBT provision such as service outreach and recovery (13), and acceptance and commitment therapy (61). This overview found psychosocial interventions generally have low retention rates, except when CM strategies are included. This finding differed from the meta-analysis contained in Minozzi et al. (37) which found no difference between CM and treatment-as-usual upon dropout rate. After assessing the included primary studies between reviews, we found that Minozzi et al. (37) included participants who used cocaine and also had severe mental health issues. Studies that focused on people who use cocaine and have severe mental disorders were included in the review by Minozzi et al. (37), but this information was outside the scope of our review. It appears differing findings arise from combining studies of people with severe mental health issues compared to studies where people do not have major psychiatric comorbidities. It may therefore be justifiable for reviews to separate studies of drug users with, and without, serious mental problems rather than treating them as one large group (30, 57, 62).

Screening for psychiatric symptoms and improving intervention participation will be important in future psychosocial intervention programs. Baker et al. (63) showed 42% of ATS dependence in the community were diagnosed with schizophrenia or schizoaffective disorder. Combining mental disorder treatment and substance abuse treatment could have positive immediate and long term effects (18).

### Interventions Targeted to Reduce HIV/AIDS Risk Behaviours

Psychosocial interventions are effective in reducing HIV/A risk behaviours. This overview identified meaningful improvements for the intervention group such as reducing HIV/AIDS risk behaviours, although of low quality of evidence. The reviews identified no significant differences between multi-section psychosocial interventions and standard education in reducing HIV risk behaviours (64, 65).

### Overall Completeness and Applicability of the Evidence

The generalisability of the evidence for interventions to reduce ATS use is a significant strength of this overview, as it included a relatively large number of primary studies and systematic reviews. The combined body of evidence enabled robust conclusions identifying the effectiveness of psychosocial intervention in reducing drug use, depression and improving retention in care. The findings of this overview might be generalisable to other types of drug use with clinically meaningful outcomes.

However, the identified studies are insufficient to address the risk of adverse outcomes for participants who also have severe mental health disorders. Further, the evidence was not sufficient to assess the effect of specific psychosocial interventions such as case management or motivational interviews. There were too few reviews and only small numbers of included studies to make a determination. There is a pressing need for further research to examine the impact of psychological therapies upon longer-term follow-up (e.g., 6, 12, and 24 months after treatment). Additionally, the body of evidence shown here underscores the need for further intervention studies in population groups of people who use ATS beyond the limited ones reported here, which primarily focused on MSM and male clients of clinics, and people with severe mental health problems who also use ATS.

### Potential Limitations and Biases in the Overview Process

A focus of this overview is investigation of the risks of biases in the present evidence-base. Drucker et al. (66) identified that potential biases in systematic reviews arise from the failure of authors to utilise standard guidelines when conducting systematic reviews, and especially in the interpretation of results. To limit potential for bias in this overview, a clear research question was framed based on PICOs framework and comprehensive searching were applied in this overview to minimise selection bias. Second, to reduce reporting bias, two reviewers independently identified, extracted data and interpreted the reviews' findings following the guidelines of conducting overviews of Cochrane (42). Although we re-analysed existing study data, we relied on identifying primary studies through existing systematic reviews and did not undertake a search for further trials. This approach is consistent with overview methodology to re-analyse data previously identified rather than search for more studies.

## Author's Conclusions

This overview combines evidence from both a narrative synthesis of systematic reviews and a new supplemental meta-analysis of their primary studies to more fully describe the current body of knowledge on the effects of psychosocial interventions on reducing ATS use and associated harms. This overview showed that psychosocial interventions are effective in reducing drug use in the short term. The evidence suggests the maintenance of treatment effects appears most achievable when psychosocial interventions are part of routine clinical practise. Further, to sustain the long-term effects, psychological treatments should include considerations of ATS uses with psychiatric comorbidities. It is also recommended that future analyses of intervention effects systematically examine the impact of case management, facilitated peer networks, and social programs to support housing and legal advice tailored for people who use ATS. Further research should focus on under-served groups of ATS users such as females, young people and people who use ATS in low- and middle-income countries.

## Implications

We found there is sufficient evidence showing psychosocial interventions can be effective, safe ways to reduce the harmful effects of ATS use on physical, mental and social well-being. To achieve the greatest benefit in reducing ATS use, it is necessary to combine different therapies and to sustain them over time to increase retention in care and improve coping skills, organise social support to help users recover and avoid high relapse situations. Clinicians and policymakers should be mindful that many studies were undertaken in high-income countries, and implementation in other settings will require further evaluation.

## Data Availability Statement

All datasets generated for this study are included in the article/Supplementary Material.

## Author Contributions

MT and QL independently searched and assessed the methodological quality of each review and wrote manuscript. GL, MD, and PB undertook a final review of included reviews. PB checked all extracted data for accuracy and consistency and revised manuscript. All authors contributed to the article and approved the submitted version.

## Conflict of Interest

The authors declare that the research was conducted in the absence of any commercial or financial relationships that could be construed as a potential conflict of interest.
