# Supplementary material for: Psychosocial Interventions for Amphetamine Type Stimulant Use Disorder: An Overview of Systematic Reviews
Source: Front Psychiatry. 2021 Jun 17;12:512076. doi: 10.3389/fpsyt.2021.512076 (PMC8245759; doi:10.3389/fpsyt.2021.512076)
Supplement: Supplementary file 1 [file Table_1.DOCX]

**Table 1: Characteristics of included systematic reviews**

| **Review title** | **Date of search/ Length of follow up** | **Population** | **Intervention** | **Comparison** | **Outcomes for which data were reported** | **Conclusion** | **Meta-Analysis/ Summary of quality of evidence in reviews (risk of bias)** | **No of included studies** |
| --- | --- | --- | --- | --- | --- | --- | --- | --- |
| 1. A systematic review of cognitive and behavioural therapies for methamphetamine dependence  **Nicole Lee & Richard Rawson (2008)** | Not mentioned | Adults who use methamphetamine | 1. CBT 2. CM 3. MI 4. Matrix | Active controls (e.g. other psychological interventions), treatment as usual (TAU) or minimal care control conditions (e.g. self-help booklets)  Only included RCT | 1. Relapse prevention: CBT may reduce methamphetamine use and other positive changes, even over very short periods of treatment (two and four sessions); CM reduces methamphetamine during the intervention, but it is not clear if these gains are sustained at post-treatment follow-up 2. Combination of MI and CBT is useful in increasing abstinence and self-efficacy to quit | 1. Psychological intervention is effective in addressing methamphetamine use and dependence.   There were no studies of newer cognitive – behavioural therapies, such as mindfulness-based cognitive therapy (MBCT), dialectical behaviour therapy (DBT) or schema therapy. | No Meta-analysis  The heterogeneity of the included studies  Authors did not:   - Comprehensively search - Measure ROB - Review independently by 2 reviewers - Evaluate heterogeneity   Following the Health evidence quality assessment tool the review is scored 6 (moderate quality) | 12 |
| 2. A review of psychological and pharmacological treatment options for methamphetamine dependence  **Sadmir Ciketic (2012);** | 1990-2009 | Adults (aged 18 years or older) with ATS dependence or abuse diagnosed by any set of criteria (DSMV, ICD-10) | Psychological treatment included: CBT, MI, CM, and a combination of different therapies | Treatment as usual or other types of treatment | 1. Outpatient and inpatient modality settings according to the level of drug use 2. Retention in treatment: moderate improvement among people who treated by CBT or counselling 3. Drug use:   psychosocial interventions are moderately effective in reducing drug use and associated problems | The overall quality of evidence was low and there was insufficient evidence | No meta-analysis   1. Heterogeneity of intervention   Insufficient controlled trials  Author did not   - Comprehensively search - Evaluate the risk of bias and GRADE of evidence - Review dependently by 2 reviewers - Evaluate heterogeneity; similar across the primary studies   Following the Health evidence quality assessment tool the review is scored 6 (moderate quality) | 7 psychological studies among 20 included studies |
| 3. When the Party is Over: A Systematic Review of Behavioral Interventions for Substance-Using Men Who Have Sex with Men  **Adam W. Carrico (2016);** | To August 2015 | Men who have sex with men and use drugs. The group of methamphetamine users is one of the targeted population groups. | 1.Cognitive and behavioural interventions  2.Motivational interviewing interventions  3.Resilience and Syndemics Interventions | Treatment as usual | 1. Substance use: Personal Cognitive Counselling decreased substance use (RR=0.51-0.93)   Risk behaviours: Reduce the number of sex partners during meth use (RR=0.26; 95% CI 0.08-0.84) | 1. Limited RCT studies and not provide evidence for the long term differential efficacy 2. Brief intervention is effective for low severity of substance use disorder symptoms   The SR did not evaluate the risk of bias of included studies | No meta-analysis  Authors did not  - Evaluate the risk of bias and GRADE of evidence  - Review dependently by 2 reviewers  - Evaluate heterogeneity; similar across the primary studies  Following the Health evidence quality assessment tool the review is scored 6 (moderate quality) | 5 studies among people who use ATS among 12 included studies |
| 4.Psychosocial interventions for psychostimulant misuse  **(Minozzi, 2016)** | 1966 to 2015 | Adults (18 years and older) with a diagnosis of psychostimulant misuse (dependence or problematic use) according to the Diagnostic and Statistical Manual of mental Disorders (DSM) III, IV or V or the ninth or tenth International Classification of diseases (ICD-9 or ICD-10) criteria, irrespective of the pattern of use, sex, age or nationality. | 1. Cognitive Behaviour Therapy (CBT) 2. Cognitive therapy 3. Coping skills training/ Relapse prevention 4. Community reinforcement approach 5. Contingency management 6. Motivational interviewing 7. Interpersonal therapy 8. Psychodynamic therapy   12 step facilitation | No treatment; treatment as usual; or alternative  psychosocial approach | 1. Dropouts: Reduced the drop out rate (RR 0.83, 95% CI; 0.76 −0.91) 2. Continuous abstinence, end of treatment: RR: 2.14; 95% CI 1.27-3.59) 3. Continuous abstinence, longest follow up: did not significantly increase RR 2.12 (0.77-5.86) 4. The longest period of abstinence: did not increase the longest period of abstinence (MD-3.15 days; 95% C -10.35;4.05) 5. Craving: SMD -0.19 (-0.48;0.1) 6. Severity of dependence: -0.48 (-1.22;0.26)   Depression: MD -4.75 (-10.45; 0.95) | 1. Drop out: moderate quality of evidence. 24 studies and 3393 participants 2. Continuous abstinence at the end of treatment: 8 studies; 1241 participants; low-quality evidence 3. Continuous abstinence, longest follow up: 4 studies, 324 participants, low-quality evidence 4. The longest period of abstinence: 1 study, 110 participants, low-quality evidence.   Psychosocial intervention probably improves treatment adherence and may increase abstinence at the end of treatment; however, people may not be able to stay clean several months after the end of treatment | Yes- meta-analysis  The reliability of this review is high (10 out of 10) but it included both people who use ATS and cocaine.  Following the Health evidence quality assessment tool the review is scored 10 (strong quality) | 11 studies among people who use ATS among 52 included studies |
| 5. Cognitive‐behavioural treatment for amphetamine‐type stimulants (ATS)‐use disorders  **Harada (2018)** | 1966- July 2018 | Adults (aged 18 years or older) with ATS dependence or abuse diagnosed by any set of criteria (DSMV, ICD-10) | Cognitive Behaviour Therapy  Type of included studies: RCT and Quasi-RCTs | Other types of psychotherapy, pharmacotherapy,  12‐step facilitation (the intervention model to promote abstinence used in the self‐help groups), no intervention or treatment as usual | 1. Abstinent days in 90 days: OR 0.22 (0.02-2.11)   Dependence symptoms (SMD -0.59 (-1.16; -0.02) | The overall quality of evidence was low and there was insufficient evidence to conclude that CBT is effective or ineffective at treating ATS use | Yes- meta analysis  The number of included studies is only 2 and among 104 participants  The comprehensive searching and heterogeneity judgement are not reliable  Following the Health evidence quality assessment tool the review is scored 8 (strong quality) | 2 |
| 6.Comparative efficacy and acceptability of psychosocial interventions for individuals with cocaine and amphetamine addiction: A systematic review and network meta-analysis  **Franco (2018)** | Between 1993 and 2016 | People who use Amphetamine or Cocaine from 18 years old or older. | 1. Contingency management +community Reinforcement Approach  2. contingency management +12 step programme  3. Community Reinforcement Approach + Non-contingent Rewards  4. Contingency management + cognitive behaviour therapy  5. Contingency management  Community reinforcement approach  6. Mediation based therapies  (mindfulness)  7.Non-contingency Rewards  8.Supportive psychodynamic therapy | Alternative interventions or TAU | 1. Drop out rate: 15.1%-60.2%. Compared to TAU OR 1.41 (1.10-1.82) 2. Abstinence at the end of treatment of Contingency management: OR=2.59 (95%CI 1.59-3.1); CM plus CBT OR 1.88 (95%CI: 2.01-6.55) 3. The longest duration of abstinence has belonged to the combination of CBT and CM (SMD 0.75 (0.31-1.19) | 1.CBT alone was more acceptable than TAU (NNT 10.5, 95% CI 5.8–53.6)  2.CM in combination with community reinforcement approach was superior to TAU for abstinence at 12 weeks of treatment (NNT 2.1, 95% CI 1.6–6.2)  3. The combined intervention, CM plus community reinforcement approach, overall achieves the best outcomes. | Yes  Heterogeneity is high, so have to run network meta-analysis  The reliability of this review is high (9 out of 10). However, comprehensive searching is not reliable because there was only 7 included studies among people who use ATS until 2018.  Following the Health evidence quality assessment tool the review is scored 9 (strong quality) | 7 studies of ATS among 50 included studies (43 studies of Cocaine) |
| 7.Methamphetamine dependence in methadone treatment services in Iran: the first literature review of a new health concern  **Zahra Alammehrjerdi (2018)** | 1 January 2005 and 28 October 2017 | Methamphetamine dependence among Iranian methadone patients | Psychosocial treatments for methamphetamine dependence and/or associated harms in Iran:   1. Family therapy 2. CBT 3. Motivational interviewing 4. Matrix model | Treatment as usual | 1. Abstinence from methamphetamine: CBT improved psychological well-being. The Matrix Model led to abstinence from methamphetamine and improved psychological well-being. Motivational interviewing increased attendance in treatment. Family therapy improved the quality of life and social support. | 1. Untreated methamphetamine dependence was associated with multiple health problems in the social and health contexts of the patients such as poor psychological wellbeing 2. No risk of bias evaluation | No   1. The number of studies was limited   Reliability of this study is moderate (7/10) because:   - The level of evidence is low when they reviewed many observation studies - They did not evaluate risk of bias or GRADE evidence - The review process is not transparent | 4 psychosocial treatment studies among 13 included studies |
| 8. Interventions to address substance use and sexual risk among gay, bisexual and other men who have sex with men who use methamphetamine: A systematic review  **Rod Knight (2019)** | To October 23, 2017 | Gay, bisexual or other men who have sex with men with a diagnosis of ATS dependence | Interventions to address/reduce MA use and associated harms and risk behaviours: 12 Pre and post and 16 RCT design  1, Motivation Interviewing (MI)  2, Contingency Management  3, Other Psychosocial interventions: Personal Cognitive Counselling | Placebo or other/no interventions | 1. Drug use 2. Sexual risk behaviours: Significant reduction in having sex while under the influence of drugs or engaging in condomless anal intercourse (CAI) | 1. Integrating interventions to address both drugs- and sexual-related harms for gbMSM who use methamphetamine can be efficacious   Most studies did not report on the differential effects of various intervention approaches by social positioning such as sexual identity, gender identity, HIV status, socioeconomic status | No   1. Heterogeneity of both intervention designs and outcome measured   Reliability of this study is strong (9/10) because:  -The narrative synthesis did not discuss consistently through discussion section from 20 psychosocial studies | 20 pyschosocial among 26 included studies |
| 9. Treatment of amphetamine abuse/use disorder: a systematic review of a recent health concern  **Mansour Khoramizadeh (2019)** | January 2001 to March 2019 | Participants were patients with high rates of daily amphetamine use and mental health problems | Any type of pharmacological treatment for an amphetamine problem was acceptable for study inclusion. BCBT needed to be conducted in agreement with the principles of Baker and colleagues’ treatment guide. BCBT refers to teaching patients to identify, evaluate and respond to their dysfunctional thoughts and beliefs and use several techniques to change thinking, mood and behaviours in less than ten sessions of psychotherapy [9] | Treatment as usual | 1. Abstinence from amphetamines had a range from 40.8% to 58.3% [20 among treated participants at six-month follow-up 2. Long-term efficacy of behavioural interventions is high in terms of treating substance abuse. High rates of treatment retention were reported at six-month and 12-month follow-ups | Abstinence from amphetamine or reduction in amphetamine abuse was confirmed in four BCBTstudies and one study which applied BCBT with a pharmacological treatment that was stable between two and 12-months. Other changes in BCBT studies were as follows: reduced polydrug use; drug injection, criminality and severity of amphetamine dependence at six-month follow-up; improved general functioning; mental health; stage of change as well as improved motivation to change in a pharmacological + BCBT study  None of the publication reported any adverse effects | No.  Self-report of drug-related problems is reliable, as long as participation is voluntary and results remain confidential  The reliability of this study is moderate (7/10) because:   - The number of included studies is small - Although the authors mentioned the risk of bias evaluation, they did not present it in the article - The method for combining results is not appropriate because the quality of each included study did not be discussed in the discussion and conclusion | 4 psychosocial studies among 10 included studies |
| 10. Psychological treatment for methamphetamine use and associated psychiatric symptom outcomes: A systematic review  **Alexandra M. Stuart (2020)** | From 2001 to 2019 | Adults who use methamphetamine | 1. CBT 2. CM 3. MI 4. Matrix | Active controls (e.g. other psychological interventions), treatment as usual (TAU) or minimal care control conditions (e.g. self-help booklets)  Only included RCT | 1. Abstinence from methamphetamine was significantly increased among the intervention conditions, as those who attended two or more sessions had higher rates of abstinence (40.8%) 2. There was a significant short-term beneficial effect of the intervention on depression, with more sessions associated with greater improvement | 1. No eligible studies were employing potentially helpful therapies such as Dialectical Behavior Therapy, Schema Therapy or Mindfulness-Based CBT, representing a gap in the literature 2. The risk of bias of individual studies was measured by Cochrane Collaboration ROB tool | No  Reliability of this study is moderate (7/10) because:   - There were a small number of randomized controlled trials examining methamphetamine and psychiatric outcomes - The method used for combining is not appropriate because the primary studies used different scales to measure depression. | 10 |
| 11. Non-pharmacological interventions for methamphetamine use disorder: a systematic review  **PV AshaRani (2020)** | January 1995 to February 2020 | Participants were clinically diagnosed (as METH abuse/dependent/use disorder) according to the Diagnostic and Statistical Manual of Mental DisorderslV/5 (International Statistical Classification of Diseases (ICD) criteria or as established by a cut-off score using the Severity of Dependence Scale (SDS) or other relevant diagnostic instruments/ criteria | Non-pharmacological interventions included: 1. Cognitive behavioural therapy (CBT),  2.Motivational interviewing (MI),  3. Exercise  4. Reinforcement-based therapies, repetitive Transcranial Magnetic Stimulation (rTMS) or other structured psychosocial interventions. | Treatment as usual/ other types of intervention | 1.Efficacy  2.Retention in treatment  3.Psychiatric symptom severity  4.Increase a cognitive function  5.Reduction in the severity of risky behaviours | 1.CM interventions among all the behavioural interventions consistently showed benefits (reduced drug use, better treatment retention, reduction in psychiatric symptoms and better quality of life) during the treatment period of METH use  2. Exercise and rTMS were also shown an effect on reducing METH use. However, small sample size + short time follow up  3. The residential programmes suggested the reduction in craving instead of drug use outcome  4. Clear evidence about Risk of bias of included studies | No meta-analysis. The reasons were explained as follow:   - Limitations: heterogeneity of included studies; - Different definitions for the same outcomes - None of the studies had population-level data - Risk of bias of included studies   The reliability of this review is strong (9/10), the heterogeneity is not measured. | 44 |
