# Supplementary material for: Psychosocial Interventions for Amphetamine Type Stimulant Use Disorder: An Overview of Systematic Reviews
Source: Front Psychiatry. 2021 Jun 17;12:512076. doi: 10.3389/fpsyt.2021.512076 (PMC8245759; doi:10.3389/fpsyt.2021.512076)
Supplement: Supplementary file 2 [file Table_2.DOCX]

**Table 2: - Reliability of included reviews (Based on Health Evidence: Quality Assessment tool)**

| **No of Reviews** | ***Clear RQ?*** | ***inclusion criteria*** | ***Compreh search*** | ***Adequate search*** | ***Level evidence*** | ***Method quality*** | ***Transparent result*** | ***Findings of results*** | ***Appro methods*** | ***Interpretation*** | **SUM** |
| --- | --- | --- | --- | --- | --- | --- | --- | --- | --- | --- | --- |
|  |  |  |  |  |  |  |  |  |  |  |  |
| 1.Lee and Rawson (2008)  Updated 2020, No14 | 1 | 1 | 1 | 0 | 1 | 0 | 0 | 0 | 1 | 1 | 6 |
| 2. Ciketic, Hayatbakhsh, Doran, Najman, and McKetin (2012); No13 | 1 | 1 | 1 | 1 | 1 | 0 | 0 | 0 | 0 | 1 | 6 |
| 3. Carrico, Zepf, Meanley, Batchelder, and Stall (2016); No 22 | 1 | 1 | 1 | 1 | 1 | 0 | 0 | 0 | 0 | 1 | 6 |
| 4.Minozzi, Saulle, De Crescenzo, and Amato (2016); No3 | 1 | 1 | 1 | 1 | 1 | 1 | 1 | 1 | 1 | 1 | 10 |
| 5. Harada, Tsutomi, Mori, and Wilson (2018); No10 | 1 | 1 | 0 | 1 | 1 | 1 | 1 | 0 | 1 | 1 | 8 |
| 6. De Crescenzo et al. (2018); No30 | 1 | 1 | 0 | 1 | 1 | 1 | 1 | 1 | 1 | 1 | 9 |
| 7. Alammehrjerdi, Ezard, and Dolan (2018); No 25 | 1 | 1 | 1 | 1 | 0 | 0 | 0 | 1 | 1 | 1 | 7 |
| 8. Knight et al. (2019)- No 18 | 1 | 1 | 1 | 1 | 1 | 1 | 1 | 1 | 0 | 1 | 9 |
| 9. Khoramizadeh et al. (2019)- No 33 | 1 | 1 | 0 | 1 | 1 | 0 | 1 | 1 | 0 | 1 | 7 |
| 10. Stuart et al. (2020)- No 31 | 1 | 1 | 0 | 1 | 1 | 1 | 1 | 1 | 1 | 1 | 8 |
| 11. AshaRani et al. (2020)- No 32 | 1 | 1 | 1 | 1 | 1 | 1 | 1 | 1 | 0 | 1 | 9 |

Alammehrjerdi, Z., Ezard, N., & Dolan, K. (2018). Methamphetamine dependence in methadone treatment services in Iran: the first literature review of a new health concern *Asian Journal of Psychiatry, 31*, 49-55. doi:<https://doi.org/10.1016/j.ajp.2018.01.001>

AshaRani, P., Hombali, A., Seow, E., Jie, W., Tan, J. H., Subramaniam, M. J. D., & Dependence, A. (2020). Non-pharmacological interventions for methamphetamine use disorder: a systematic review. 108060.

Carrico, A. W., Zepf, R., Meanley, S., Batchelder, A., & Stall, R. (2016). Critical Review: When the Party is Over: A Systematic Review of Behavioral Interventions for Substance-Using Men Who Have Sex with Men *Journal of acquired immune deficiency syndromes (1999), 73*(3), 299. doi:10.1097/QAI.0000000000001102

Ciketic, S., Hayatbakhsh, M. R., Doran, C. M., Najman, J. M., & McKetin, R. (2012). A review of psychological and pharmacological treatment options for methamphetamine dependence *Journal of Substance Use, 17*(4), 363-383. doi:10.3109/14659891.2011.592900

De Crescenzo, F., Ciabattini, M., D’Alò, G. L., De Giorgi, R., Del Giovane, C., Cassar, C., . . . Cipriani, A. (2018). Comparative efficacy and acceptability of psychosocial interventions for individuals with cocaine and amphetamine addiction: A systematic review and network meta-analysis. *PLoS Medicine, 15*(12), e1002715. doi:10.1371/journal.pmed.1002715

Harada, T., Tsutomi, H., Mori, R., & Wilson, D. B. (2018). Cognitive‐behavioural treatment for amphetamine‐type stimulants (ATS)‐use disorders *Cochrane Database of Systematic Reviews*(12). doi:10.1002/14651858.CD011315.pub2

Khoramizadeh, M., Effatpanah, M., Mostaghimi, A., Rezaei, M., Mahjoub, A., & Shishehgar, S. J. D. J. o. P. S. (2019). Treatment of amphetamine abuse/use disorder: a systematic review of a recent health concern. 1.

Knight, R., Karamouzian, M., Carson, A., Edward, J., Carrieri, P., Shoveller, J., . . . Fast, D. (2019). Interventions to address substance use and sexual risk among gay, bisexual and other men who have sex with men who use methamphetamine: A systematic review. *Drug and Alcohol Dependence, 194*, 410-429. doi:<https://doi.org/10.1016/j.drugalcdep.2018.09.023>

Lee, N. K., & Rawson, R. A. (2008). A systematic review of cognitive and behavioural therapies for methamphetamine dependence *Drug Alcohol Rev, 27*(3), 309-317. doi:10.1080/09595230801919494

Minozzi, S., Saulle, R., De Crescenzo, F., & Amato, L. (2016). Psychosocial interventions for psychostimulant misuse *Cochrane Database of Systematic Reviews*(9). doi:10.1002/14651858.CD011866.pub2

Stuart, A. M., Baker, A. L., Denham, A. M. J., Lee, N. K., Hall, A., Oldmeadow, C., . . . McCarter, K. (2020). Psychological treatment for methamphetamine use and associated psychiatric symptom outcomes: A systematic review. *Journal of substance abuse treatment, 109*, 61-79. doi:<https://doi.org/10.1016/j.jsat.2019.09.005>
