# Supplementary figures and images for: Psychosocial Interventions for Amphetamine Type Stimulant Use Disorder: An Overview of Systematic Reviews
Source: Front Psychiatry. 2021 Jun 17;12:512076. doi: 10.3389/fpsyt.2021.512076 (PMC8245759; doi:10.3389/fpsyt.2021.512076)

**Table 3: Risk of bias of included primary studies**


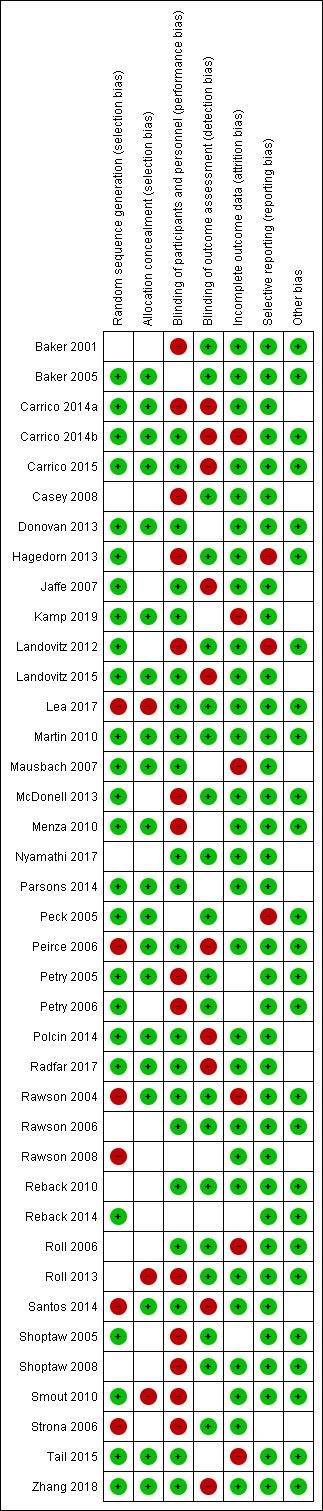

Supplement: Supplementary file 3 [file Table_3.DOCX]
