# Supplementary material for: Psychosocial Interventions for Amphetamine Type Stimulant Use Disorder: An Overview of Systematic Reviews
Source: Front Psychiatry. 2021 Jun 17;12:512076. doi: 10.3389/fpsyt.2021.512076 (PMC8245759; doi:10.3389/fpsyt.2021.512076)
Supplement: Supplementary file 4 [file Table_4.DOCX]

**Table 4: The different tools used in primary studies which measured the effect of psychosocial interventions among people who use ATS**

| **Measure** | **Tools** |
| --- | --- |
| 1. Drug use | **Drug use**  1. Opiate Treatment Index (OTI, Darke 1992)  2. Stimulant Use: Self -reported the number of days they used methamphetamine in the past 30 days; a Urine sample  3. Self- report substance use: Addiction Severity Index (ASI) composite scores (1) (2) (3) (4)  4. Drug use: method of use; drug acquisition, injecting drug use, high-risk behaviours, drug-related problems and treatment access  5. Minnesota Cocaine Craving Scale 0-10 (strong) -17 Martin  6. Substance use: ASI lite (past 30 days) + additional questions assessed meth use in past 3 months, including the number of discrete crystal methamphetamine episodes.; the frequency of other substances used in the past 3 months in the context of sex (using a few hours prior to or during sex) (5)  9. Injection drugs: Behavioural Questionnaire Amphetamine (Reback 2007) (1)  10. Number of day drug: TLFB (Sobell 1993) (6)  11. Biological test (urine, hair): (6) (7) (3) (4)  12. ATS use and polydrug use: ASSIST (8)  **The severity of drug use**   1. The amphetamine version of the Severity of Dependence Scale (SDS, Gossop 1995) -(9)*, 13 Martin, (10)* 2. Drug dependence: DSM-IV- diagnoses of ecstasy abuse and dependence (GAIN; Dennis 1999) (1) (6) (2) (3) 3. Severe dependence: Severity of dependence: Leeds dependence questionnaire (LDQ) (11) |
| 1. Stage of change | 1. Refusal self-efficacy: Amphetamine Refusal Self Efficacy Questionnaire (ARSEQ)- Sitharthan, Job, Kavanagh, Sitharthan and Hough (2003) 2. Engagement in positive events: The Behavioural Activation Scale (BADS) (5) 3. Change in information, motivation and behavioural skills (IMB models of Fishers) 4. General motivational measures: readiness to change questionnaire; Commitment to Abstinence Scale, and Worker Alliance Inventory short form, therapy-specific measures (acceptance and action questionnaire) (11) 5. Contemplation ladder (Biener &Abrams,1991) 6. Positive and negative affect: Adapted version of the Differential Emotions Scale (DES)   7. Help-seeking intention: General Health seeking questionnaire (Wilson, 2005)  8. Actual help seeking (Actual help seeking questionnaire) |
| 1. Risk behaviours | 1. Sexual risk-taking behaviours: a. number of anal sex partners in the past 3 months; b. Number of risky and sex partners on methamphetamine 2. Behavioural Questionnaire-Amphetamine: BQ-A (Chesney, Chambers & Kahn,1997) (4) 3. Self-efficacy for condom use – 15 Mausbach (12) 4. Self-efficacy for negotiating safer sex – 15 Mausbach (12) 5. Unprotected anal intercourse with a partner of unknown or discordant HIV status (non-concordant UAI) – (13) (6) 6. The number of non-concordant UAI partners (13) 7. HIV sexual risk behaviour: number of episodes of unprotected anal intercourse in the past 3 months; self-perceived risk for HIV infection on 10 point scale (5) 8. Critical life events: vacations, birthdays, paycheck days, parties (6) 9. Knowledge, Option and Attitude towards HIV/AIDS: BSS questionnaire (14) 10. Oral HIV test (7) |
| 1. Retention in care | 1. General social functioning: Brief treatment outcome measure (BTOM; Lawrinson, Copeland & Indig,2005) – 13 Martin 2. Attendance rate: (2) 3. Successful linkage to services (15) (16) (17) 4. Treatment adherence (16) 5. Link to community services (15) 6. Lost to treatment (18) 7. Drop out (19) (20) 8. Retention follow up: 0-3 months, six months (21) |
| 1. Mental health | 1. General Health Questionnaire (GHQ-28) - Goldberg & Hillier,1979: a. Somatic symptoms, b. Anxiety; c. Social dysfunction; d. depression 2. Mental health : Brief symptom Inventory (BSI) - Derogatis & Melisaratos 1983 3. The Beck Depression Inventory I, II (BDI-I, II) - beck 1988 (6) (2) (11) 4. International Personality Disorder Examination Questionnaire (IPDEQ) 5. Psychological distress: Kessler Psychological distress Scale (K10) in the previous 4 weeks -12 Lea 6. Depressive symptoms: Montgomery - Asberg Depression Rating Scales (MADRS) (5) 7. Internalized Homophobia (1) 8. Depressive symptomatology: CESD, Radloff 1977 (1) |
| 1. Reduce crime | 1. Arrests (22) (23) 2. Recidivism (22) (23) 3. Legal problems (15) 4. Criminal activities in the last 12 months (23) |
| 1. Quality of life | 1. Short-form survey (SF-36) with 8 dimensions: physical functioning, social functioning, role limitation, bodily pain, mental health, vitality and general health 2. Quality of life: the 8 items European Health Interview Survey- Quality of Life (EURO-HIS-QOL) – (9), (8) |
| 1. Social network | 1. ACASI: Demographic characteristics; drug and alcohol use, injection patterns, sexual risk behaviours, mental health status, social network, social support, health beliefs and self-efficacy (7).(10) 2. Employment score (15) (23) 3. Change in one relationship (24) |

1. Nyamathi A, Reback CJ, Shoptaw S, Salem BE, Zhang S, Yadav K. Impact of Tailored Interventions to Reduce Drug Use and Sexual Risk Behaviors Among Homeless Gay and Bisexual Men. American journal of men's health. 2017;11(2):208-20.

2. Rawson RA, Marinelli-Casey P, Anglin MD, Dickow A, Frazier Y, Gallagher C, et al. A multi-site comparison of psychosocial approaches for the treatment of methamphetamine dependence. Addiction. 2004;99(6):708-17.

3. Roll JM, Petry NM, Stitzer ML, Brecht ML, Peirce JM, McCann MJ, et al. Contingency management for the treatment of methamphetamine use disorders. The American journal of psychiatry. 2006;163(11):1993-9.

4. Reback CJ, Shoptaw S. Development of an evidence-based, gay-specific cognitive behavioral therapy intervention for methamphetamine-abusing gay and bisexual men. Addictive behaviors. 2014;39(8):1286-91.

5. Mimiaga MJ, Reisner SL, Pantalone DW, O'Cleirigh C, Mayer KH, Safren SA. A pilot trial of integrated behavioral activation and sexual risk reduction counseling for HIV-uninfected men who have sex with men abusing crystal methamphetamine. AIDS Patient Care STDS. 2012;26(11):681-93.

6. Parsons JT, Lelutiu-Weinberger C, Botsko M, Golub SA. A randomized controlled trial utilizing motivational interviewing to reduce HIV risk and drug use in young gay and bisexual men. J Consult Clin Psychol. 2014;82(1):9-18.

7. Reback CJ, Grant DL, Fletcher JB, Branson CM, Shoptaw S, Bowers JR, et al. Text messaging reduces HIV risk behaviors among methamphetamine-using men who have sex with men. AIDS Behav. 2012;16(7):1993-2002.

8. Tait RJ, McKetin R, Kay-Lambkin F, Carron-Arthur B, Bennett A, Bennett K, et al. Six-month outcomes of a Web-based intervention for users of amphetamine-type stimulants: randomized controlled trial. Journal of medical Internet research. 2015;17(4):e105.

9. Lea T, Kolstee J, Lambert S, Ness R, Hannan S, Holt M. Methamphetamine treatment outcomes among gay men attending a LGBTI-specific treatment service in Sydney, Australia. PLoS One. 2017;12(2):e0172560.

10. Santos GM, Coffin PO, Vittinghoff E, DeMicco E, Das M, Matheson T, et al. Substance use and drinking outcomes in Personalized Cognitive Counseling randomized trial for episodic substance-using men who have sex with men. Drug Alcohol Depend. 2014;138:234-9.

11. Smout MF, Longo M, Harrison S, Minniti R, Wickes W, White JM. Psychosocial treatment for methamphetamine use disorders: a preliminary randomized controlled trial of cognitive behavior therapy and Acceptance and Commitment Therapy. Substance abuse. 2010;31(2):98-107.

12. Mausbach BT, Semple SJ, Strathdee SA, Zians J, Patterson TL. Efficacy of a behavioral intervention for increasing safer sex behaviors in HIV-positive MSM methamphetamine users: results from the EDGE study. Drug Alcohol Depend. 2007;87(2-3):249-57.

13. Menza TW, Jameson DR, Hughes JP, Colfax GN, Shoptaw S, Golden MR. Contingency management to reduce methamphetamine use and sexual risk among men who have sex with men: a randomized controlled trial. BMC Public Health. 2010;10:774.

14. Radfar SR, Mohsenifar S, Noroozi A. Integration of Methamphetamine Harm Reduction into Opioid Harm Reduction Services in Iran: Preliminary Results of a Pilot Study. 2017;11(2):e7730.

15. Hesse M, Vanderplasschen W, Rapp RC, Broekaert E, Fridell M. Case management for persons with substance use disorders. The Cochrane database of systematic reviews. 2007(4):Cd006265.

16. Martinez-Vispo C, Martinez U, Lopez-Duran A, Fernandez Del Rio E, Becona E. Effects of behavioural activation on substance use and depression: a systematic review Subst Abuse Treat Prev Policy. 2018;13(1):36.

17. Joo JY, Huber DL. Community-based case management effectiveness in populations that abuse substances International nursing review. 2015;62(4):536-46.

18. Hunt GE, Siegfried N, Morley K, Sitharthan T, Cleary M. Psychosocial interventions for people with both severe mental illness and substance misuse. Cochrane Database of Systematic Reviews. 2013(10).

19. Minozzi S, Saulle R, De Crescenzo F, Amato L. Psychosocial interventions for psychostimulant misuse Cochrane Database of Systematic Reviews. 2016(9).

20. Knapp WP, Soares BGO, Farrell MF, Silva de Lima M. Psychosocial interventions for cocaine and psychostimulant amphetamines related disorders Cochrane Database of Systematic Reviews. 2015(4).

21. Smedslund G, Berg RC, Hammerstrøm KT, Steiro A, Leiknes KA, Dahl HM, et al. Motivational interviewing for substance abuse Cochrane Database of Systematic Reviews. 2011(5).

22. Perry AE, Neilson M, Martyn‐St James M, Glanville JM, Woodhouse R, Hewitt C. Interventions for female drug‐using offenders Cochrane Database of Systematic Reviews. 2015(6).

23. Smith LA, Gates S, Foxcroft D. Therapeutic communities for substance related disorder Cochrane Database of Systematic Reviews. 2006(1).

24. Chiesa A, Serretti A. Are Mindfulness-Based Interventions Effective for Substance Use Disorders? A Systematic Review of the Evidence Substance Use & Misuse. 2014;49(5):492-512.
