# Supplementary material for: Psychosocial Interventions for Amphetamine Type Stimulant Use Disorder: An Overview of Systematic Reviews
Source: Front Psychiatry. 2021 Jun 17;12:512076. doi: 10.3389/fpsyt.2021.512076 (PMC8245759; doi:10.3389/fpsyt.2021.512076)
Supplement: Supplementary file 5 [file Table_5.DOCX]

**Table 5: Number primary studies followed by types of interventions**

| **Intervention type** | **Number of primary studies** |
| --- | --- |
| 1. Cognitive behaviour therapy (CBT)/ Cognitive Behaviour Therapy combined with Text messages/ Computer-based Cognitive Behaviour Therapy intervention | 19 |
| 1. Contingency management (CM) | 14 |
| 1. Psychosocial therapies (combined CBT+ MI+ CM+BI + others); Matrix | 10 |
| 1. Motivational interviewing (MI) | 7 |
| 1. Brief intervention (BI) | 4 |
| 1. Case management | 2 |
| 1. Community-based management, therapeutic community; harm reduction group therapy | 6 |
| 1. Mindfulness | 2 |
| 1. 12 steps facilitation | 1 |
| 1. Family therapy | 2 |
| SUM | 67 |

1. **Cognitive Behaviour Therapy**
2. Abdoli, N., Farnia, V., Salemi, S., Tatari, F., Juibari, T. A., Alikhani, M., & Basanj, B. (2019). Efficacy of the Marlatt cognitive-behavioral model on decreasing relapse and craving in women with methamphetamine dependence: A clinical trial. *Journal of Substance Use, 24*(2), 229-232. doi:10.1080/14659891.2018.1549279
3. Baker, A., Boggs, T. G., & Lewin, T. J. (2001). Randomized controlled trial of brief cognitive-behavioural interventions among regular users of amphetamine. *96*(9), 1279-1287. doi:<https://doi.org/10.1046/j.1360-0443.2001.96912797.x>
4. Baker, A., Lee, N. K., Claire, M., Lewin, T. J., Grant, T., Pohlman, S., . . . Carr, V. J. (2005). Brief cognitive behavioural interventions for regular amphetamine users: a step in the right direction. *100*(3), 367-378. doi:<https://doi.org/10.1111/j.1360-0443.2005.01002.x>
5. Brooks, S. J., Wiemerslage, L., Burch, K. H., Maiorana, S. A., Cocolas, E., Schiöth, H. B., . . . Stein, D. J. (2017). The impact of cognitive training in substance use disorder: the effect of working memory training on impulse control in methamphetamine users. *Psychopharmacology (Berlin, Germany), 234*(12), 1911-1921. doi:10.1007/s00213-017-4597-6
6. Feeney, G. F. X., Connor, J. P., Young, R. M., Tucker, J., & McPherson, A. (2006). Improvement in measures of psychological distress amongst amphetamine misusers treated with brief cognitiveubehavioural therapy. *Addictive behaviors, 31*(3), 1833.
7. Kamp, F., Proebstl, L., Hager, L., Schreiber, A., Riebschläger, M., Neumann, S., . . . Koller, G. (2019). Effectiveness of methamphetamine abuse treatment: Predictors of treatment completion and comparison of two residential treatment programs. *Drug and Alcohol Dependence, 201*, 8-15. doi:10.1016/j.drugalcdep.2019.04.010
8. Lea, T., Kolstee, J., Lambert, S., Ness, R., Hannan, S., & Holt, M. (2017). Methamphetamine treatment outcomes among gay men attending a LGBTI-specific treatment service in Sydney, Australia. *PLoS ONE, 12*(2), e0172560-e0172560. doi:10.1371/journal.pone.0172560
9. Mausbach, B. T., Semple, S. J., Strathdee, S. A., Zians, J., & Patterson, T. L. (2007). Efficacy of a behavioral intervention for increasing safer sex behaviors in HIV-positive MSM methamphetamine users: Results from the EDGE study. *Drug and Alcohol Dependence, 87*(2), 249-257. doi:10.1016/j.drugalcdep.2006.08.026
10. Mimiaga, M. J., Reisner, S. L., Pantalone, D. W., O'Cleirigh, C., Mayer, K. H., & Safren, S. A. (2012). A Pilot Trial of Integrated Behavioral Activation and Sexual Risk Reduction Counseling for HIV-Uninfected Men Who Have Sex with Men Abusing Crystal Methamphetamine. *AIDS Patient Care and STDs, 26*(11), 681-693. doi:10.1089/apc.2012.0216
11. Mimiaga, M. J., Pantalone, D. W., Biello, K. B., Hughto, J. M. W., Frank, J., O'Cleirigh, C., . . . Safren, S. A. (2019). An initial randomized controlled trial of behavioral activation for treatment of concurrent crystal methamphetamine dependence and sexual risk for HIV acquisition among men who have sex with men. *AIDS Care, 31*(9), 1083-1095. doi:10.1080/09540121.2019.1595518
12. Reback, C. J., & Fletcher, J. B. (2017). Outcomes from a homegrown HIV prevention program for extremely high-risk, substance-using men who have sex with men with multiple health disparities. *Journal of Gay & Lesbian Social Services, 29*(2), 167-181. doi:10.1080/10538720.2017.1296394
13. Santos, G.-M., Coffin, P. O., Vittinghoff, E., DeMicco, E., Das, M., Matheson, T., . . . Dilley, J. W. (2014). Substance use and drinking outcomes in Personalized Cognitive Counseling randomized trial for episodic substance-using men who have sex with men. *Drug and Alcohol Dependence, 138*, 234-239. doi:10.1016/j.drugalcdep.2014.02.015
14. Shoptaw, S., Reback, C. J., Peck, J. A., Yang, X., Rotheram-Fuller, E., Larkins, S., . . . Hucks-Ortiz, C. (2005). Behavioral treatment approaches for methamphetamine dependence and HIV-related sexual risk behaviors among urban gay and bisexual men. *Drug and Alcohol Dependence, 78*(2), 125-134. doi:10.1016/j.drugalcdep.2004.10.004
15. Shoptaw, S. P. D., Reback, C. J. P. D., Larkins, S. P. D., Wang, P.-C. P. D., Rotheram-Fuller, E. P. D., Dang, J. M. A., & Yang, X. P. D. (2008). Outcomes using two tailored behavioral treatments for substance abuse in urban gay and bisexual men. *Journal of substance abuse treatment, 35*(3), 285-293. doi:10.1016/j.jsat.2007.11.004
16. Smout, M. F., Longo, M., Harrison, S., Minniti, R., Wickes, W., & White, J. M. (2010). Psychosocial Treatment for Methamphetamine Use Disorders: A Preliminary Randomized Controlled Trial of Cognitive Behavior Therapy and Acceptance and Commitment Therapy. *Subst Abus, 31*(2), 98-107. doi:10.1080/08897071003641578

**Cognitive Behaviour Therapy combined with Text messages**

1. Reback, C. J., Grant, D. L., Fletcher, J. B., Branson, C. M., Shoptaw, S., Bowers, J. R., . . . Mansergh, G. (2012). Text Messaging Reduces HIV Risk Behaviors Among Methamphetamine-Using Men Who Have Sex with Men. *AIDS and Behavior, 16*(7), 1993-2002. doi:10.1007/s10461-012-0200-7
2. Reback, C. J., Fletcher, J. B., Swendeman, D. A., & Metzner, M. (2019). Theory-Based Text-Messaging to Reduce Methamphetamine Use and HIV Sexual Risk Behaviors Among Men Who Have Sex with Men: Automated Unidirectional Delivery Outperforms Bidirectional Peer Interactive Delivery. *AIDS and Behavior, 23*(1), 37-47. doi:10.1007/s10461-018-2225-z

**Computer-based Cognitive Behaviour Therapy intervention**

1. Manning, V., Garfield, J. B. B., Mroz, K., Campbell, S. C., Piercy, H., Staiger, P. K., . . . Verdejo-Garcia, A. (2019). Feasibility and acceptability of approach bias modification during methamphetamine withdrawal and related methamphetamine use outcomes. *Journal of substance abuse treatment, 106*, 12-18. doi:10.1016/j.jsat.2019.07.008
2. Tait, R. J., McKetin, R., Kay-Lambkin, F., Carron-Arthur, B., Bennett, A., Bennett, K., . . . Griffiths, K. M. (2015). Six-month outcomes of a Web-based intervention for users of amphetamine-type stimulants: randomized controlled trial. *J Med Internet Res, 17*(4), e105-e105. doi:10.2196/jmir.3778
3. **Contingency management**
4. Chudzynski, J., Roll, J. M., McPherson, S., Cameron, J. M., & Howell, D. N. (2015). Reinforcement Schedule Effects on Long-Term Behavior Change. *The Psychological record, 65*(2), 347-353. doi:10.1007/s40732-014-0110-3
5. Fletcher, J. B., Dierst-Davies, R., & Reback, C. J. (2014). Contingency management voucher redemption as an indicator of delayed gratification. *Journal of substance abuse treatment, 47*(1), 73-77. doi:10.1016/j.jsat.2014.03.003
6. Hagedorn, H. J. P. D., Noorbaloochi, S. P. D., Simon, A. B. M. S., Bangerter, A. B. S., Stitzer, M. L. P. D., Stetler, C. B. P. D. R. N., & Kivlahan, D. P. D. (2013). Rewarding early abstinence in Veterans Health Administration addiction clinics. *Journal of substance abuse treatment, 45*(1), 109-117. doi:10.1016/j.jsat.2013.01.006
7. Landovitz, R. J., Fletcher, J. B., Inzhakova, G., Lake, J. E., Shoptaw, S., & Reback, C. J. (2012). A Novel Combination HIV Prevention Strategy: Post-Exposure Prophylaxis with Contingency Management for Substance Abuse Treatment Among Methamphetamine-Using Men Who Have Sex with Men. *AIDS Patient Care and STDs, 26*(6), 32-328. doi:10.1089/apc.2011.0432
8. Landovitz, R. J., Fletcher, J. B., Shoptaw, S., & Reback, C. J. (2015). Contingency management facilitates the use of postexposure prophylaxis among stimulant-using men who have sex with men. *Open forum infectious diseases, 2*(1), ofu114-ofu114. doi:10.1093/ofid/ofu114
9. Menza, T. W., Jameson, D. R., Hughes, J. P., Colfax, G. N., Shoptaw, S., & Golden, M. R. (2010). Contingency management to reduce methamphetamine use and sexual risk among men who have sex with men: a randomized controlled trial. *BMC Public Health, 10*(1), 774. doi:10.1186/1471-2458-10-774
10. Okafor, C. N., Stein, D. J., Dannatt, L., Ipser, J., Nunen, L. J., Lake, M. T., . . . Shoptaw, S. (2019). Contingency management treatment for methamphetamine use disorder in South Africa. *Drug and Alcohol Review, 39*(3), 216-222. doi:10.1111/dar.13019
11. Peirce, J. M., Petry, N. M., Stitzer, M. L., Blaine, J., Kellogg, S., Satterfield, F., . . . Li, R. (2006). Effects of Lower-Cost Incentives on Stimulant Abstinence in Methadone Maintenance Treatment: A National Drug Abuse Treatment Clinical Trials Network Study. *Archives of general psychiatry, 63*(2), 201-208.
12. Petry, N. M., Peirce, J. M., Stitzer, M. L., Blaine, J., Roll, J. M., Cohen, A., . . . Li, R. (2005). Effect of prize-based incentives on outcomes in stimulant abusers in outpatient psychosocial treatment programs: a national drug abuse treatment clinical trials network study. *Arch Gen Psychiatry, 62*(10), 1148-1156. doi:10.1001/archpsyc.62.10.1148doi:10.1001/archpsyc.63.2.201
13. Reback, C. J. P. D., Peck, J. A. P. D., Dierst-Davies, R. M. P. H., Nuno, M. P. D., Kamien, J. B. P. D., & Amass, L. P. D. (2010). Contingency management among homeless, out-of-treatment men who have sex with men. *Journal of substance abuse treatment, 39*(3), 255-263. doi:10.1016/j.jsat.2010.06.007
14. Roll, J. M., Chudzynski, J., Cameron, J. M., Howell, D. N., & McPherson, S. (2013). Duration effects in contingency management treatment of methamphetamine disorders. *Addictive behaviors, 38*(9), 2455-2462. doi:10.1016/j.addbeh.2013.03.018
15. Roll, J. M., Petry, N. M., Stitzer, M. L., Brecht, M. L., Peirce, J. M., McCann, M. J., . . . Kellogg, S. (2006). Contingency Management for the Treatment of Methamphetamine Use Disorders. *American Journal of Psychiatry, 163*(11), 1993-1999. doi:10.1176/ajp.2006.163.11.1993
16. Strona, F. V., McCright, J., Hjord, H., Ahrens, K., Tierney, S., Shoptaw, S., & Klausner, J. D. (2006). The Acceptability and Feasibility of the Positive Reinforcement Opportunity Project, a Community-Based Contingency Management Methamphetamine Treatment Program for Gay and Bisexual Men in San Francisco. *Journal of psychoactive drugs, 38*(sup3), 377-383. doi:10.1080/02791072.2006.10400601
17. McDonell, M. G., Srebnik, D., Angelo, F., McPherson, S., Lowe, J. M., Sugar, A., . . . Ries, R. K. (2013). Randomized Controlled Trial of Contingency Management for Stimulant Use in Community Mental Health Patients With Serious Mental Illness. *Am J Psychiatry, 170*(1), 94-101. doi:10.1176/appi.ajp.2012.11121831
18. **Combination therapy, Matrix**
19. Amiri, Z., Mirzaee, B., Sabet, M. J. I. J. o. M. R., & Sciences, H. (2016). Evaluating the efficacy of Regulated 12-Session Matrix Model in reducing susceptibility in methamphetamine-dependent individuals. *5*(2), 77-85.
20. Jaffe, A., Shoptaw, S., Stein, J. A., Reback, C. J., & Rotheram-Fuller, E. (2007). Depression Ratings, Reported Sexual Risk Behaviors, and Methamphetamine Use: Latent Growth Curve Models of Positive Change Among Gay and Bisexual Men in an Outpatient Treatment Program. *Exp Clin Psychopharmacol, 15*(3), 301-307. doi:10.1037/1064-1297.15.3.301
21. Ling Murtaugh, K., Krishnamurti, T., Davis, A. L., Reback, C. J., & Shoptaw, S. (2013). Spend Today, Clean Tomorrow: Predicting Methamphetamine Abstinence in a Randomized Controlled Trial. *Health psychology, 32*(9), 958-966. doi:10.1037/a0032922
22. Marinelli-Casey, P. P. D., Gonzales, R. M. P. H., Hillhouse, M. P. D., Ang, A. P. D., Zweben, J. P. D., Cohen, J. P. D. M. P. H., . . . Rawson, R. A. P. D. (2008). Drug court treatment for methamphetamine dependence: Treatment response and posttreatment outcomes. *Journal of substance abuse treatment, 34*(2), 242-248. doi:10.1016/j.jsat.2007.04.005
23. Nyamathi, A., Reback, C. J., Shoptaw, S., Salem, B. E., Zhang, S., & Yadav, K. (2017). Impact of Tailored Interventions to Reduce Drug Use and Sexual Risk Behaviors Among Homeless Gay and Bisexual Men. *Am J Mens Health, 11*(2), 208-220. doi:10.1177/155798831559083
24. Peck, J. A. (2005). Sustained Reductions in Drug Use and Depression Symptoms from Treatment for Drug Abuse in Methamphetamine-Dependent Gay and Bisexual Men. *Journal of urban health, 82*(1_suppl_1), i100-i108. doi:10.1093/jurban/jti029
25. Rawson, R. A., Marinelli-Casey, P., Anglin, M. D., Dickow, A., Frazier, Y., Gallagher, C., . . . Zweben, J. (2004). A multi-site comparison of psychosocial approaches for the treatment of methamphetamine dependence. *Addiction (Abingdon, England), 99*(6), 708-717. doi:10.1111/j.1360-0443.2004.00707.x
26. Rawson, R. A., McCann, M. J., Flammino, F., Shoptaw, S., Miotto, K., Reiber, C., & Ling, W. (2006). A comparison of contingency management and cognitive-behavioral approaches for stimulant-dependent individuals. *101*(2), 267-274. doi:<https://doi.org/10.1111/j.1360-0443.2006.01312.x>
27. Rawson, R. A. P. D., Gonzales, R. P. D., Pearce, V. M. P. H., Ang, A. P. D., Marinelli-Casey, P. P. D., & Brummer, J. B. A. (2008). Methamphetamine dependence and human immunodeficiency virus risk behavior. *Journal of substance abuse treatment, 35*(3), 279-284. doi:10.1016/j.jsat.2007.11.003
28. Reback, C. J., & Shoptaw, S. (2014). Development of an evidence-based, gay-specific cognitive behavioral therapy intervention for methamphetamine-abusing gay and bisexual men. *Addictive behaviors, 39*(8), 1286-1291. doi:10.1016/j.addbeh.2011.11.029
29. **Motivational Interviewing**
30. Baker, A., Lewin, T., Reichler, H., Clancy, R., Carr, V., Garrett, R., . . . Terry, M. (2002). Evaluation of a motivational interview for substance use within psychiatric in-patient services. *Addiction, 97*(10), 1329-1337. doi:10.1046/j.1360-0443.2002.00178.x
31. Galloway, G. P., Polcin, D., Kielstein, A., Brown, M., & Mendelson, J. (2007). A Nine Session Manual of Motivational Enhancement Therapy for Methamphetamine Dependence: Adherence and Efficacy. *Journal of psychoactive drugs, 39*(sup4), 393-400. doi:10.1080/02791072.2007.10399900
32. Korcha, R. A. M. A., Polcin, D. L. E. D., Evans, K. B. A., Bond, J. C. P. D., & Galloway, G. P. P. D. (2013). Intensive Motivational Interviewing for women with concurrent alcohol problems and methamphetamine dependence. *Journal of substance abuse treatment, 46*(2), 113-119. doi:10.1016/j.jsat.2013.08.013
33. Marsden, J., Stillwell, G., Barlow, H., Boys, A., Taylor, C., Hunt, N., & Farrell, M. (2006). An evaluation of a brief motivational intervention among young ecstasy and cocaine users: no effect on substance and alcohol use outcomes. *Addiction, 101*(7), 1014-1026. doi:10.1111/j.1360-0443.2006.01290.x
34. Parsons, J. T., Lelutiu-Weinberger, C., Botsko, M., & Golub, S. A. (2014). A Randomized Controlled Trial Utilizing Motivational Interviewing to Reduce HIV Risk and Drug Use in Young Gay and Bisexual Men. *Journal of Consulting and Clinical Psychology, 82*(1), 9-18. doi:10.1037/a0035311
35. Polcin, D. L., Bond, J., Korcha, R., Nayak, M. B., Galloway, G. P., & Evans, K. (2014). Randomized Trial of Intensive Motivational Interviewing for Methamphetamine Dependence. *Journal of Addictive Diseases, 33*(3), 253-265. doi:10.1080/10550887.2014.950029
36. Zule, W. A., Poulton, W. E., Coomes, C. M., Mansergh, G., Charania, M., Wechsberg, W. M., & Jones, H. E. (2012). Results of a Pilot Study to Reduce Methamphetamine Use and Sexual Risk Behaviors Among Methamphetamine-Using Men Who Have Sex with Men (MSM) Not Currently in Treatment. *Journal of psychoactive drugs, 44*(5), 351-358. doi:10.1080/02791072.2012.736794
37. **Brief Intervention**
38. Galai, N., Sirirojn, B., Aramrattana, A., Srichan, K., Thomson, N., Golozar, A., . . . Celentano, D. D. (2018). A cluster randomized trial of community mobilization to reduce methamphetamine use and HIV risk among youth in Thailand: Design, implementation and results. *Soc Sci Med, 211*, 216-223. doi:10.1016/j.socscimed.2018.06.013
39. Martin, G., & Copeland, J. (2010). Brief intervention for regular ecstasy (MDMA) users: Pilot randomized trial of a Check-up model. *Journal of Substance Use, 15*(2), 131-142. doi:10.3109/14659890903075074
40. Srisurapanont, M., Sombatmai, S., & Boripuntakul, T. (2007). Brief Intervention for Students with Methamphetamine Use Disorders: A Randomized Controlled Trial. *The American journal on addictions, 16*(2), 111-116. doi:10.1080/10550490601184431
41. Zhang, S. X., Shoptaw, S., Reback, C. J., Yadav, K., & Nyamathi, A. M. (2018). Cost-effective way to reduce stimulant-abuse among gay/bisexual men and transgender women: a randomized clinical trial with a cost comparison. *Public health (London), 154*, 151-160. doi:10.1016/j.puhe.2017.10.024
42. **Case management**
43. Nyamathi, A. M., Salem, B. E., Farabee, D., & Zhang, S. (2017). Differential reporting of drug use among gay, bisexual and transgender stimulant-using homeless adults post intervention. *Journal of Substance Use, 22*(2), 218-224. doi:10.1080/14659891.2016.1179805
44. Zhang, S. X., Shoptaw, S., Reback, C. J., Yadav, K., & Nyamathi, A. M. (2018). Cost-effective way to reduce stimulant-abuse among gay/bisexual men and transgender women: a randomized clinical trial with a cost comparison. *Public health (London), 154*, 151-160. doi:10.1016/j.puhe.2017.10.024
45. **Community-based management**
46. Carrico, A. W., Flentje, A., Gruber, V. A., Woods, W. J., Discepola, M. V., Dilworth, S. E., . . . Siever, M. D. (2014). Community-based harm reduction substance abuse treatment with methamphetamine-using men who have sex with men. *J Urban Health, 91*(3), 555-567. doi:10.1007/s11524-014-9870-y
47. Dean, A. C., Dean, A. C., Nurmi, E. L., Nurmi, E. L., Moeller, S. J., Moeller, S. J., . . . London, E. D. (2019). No effect of attentional bias modification training in methamphetamine users receiving residential treatment. *Psychopharmacology, 236*(2), 709-721. doi:10.1007/s00213-018-5100-8
48. Maglione, M., Chao, B., & Anglin, M. D. (2000). Correlates of Outpatient Drug Treatment Drop-Out Among Methamphetamine Users. *Journal of psychoactive drugs, 32*(2), 221-228. doi:10.1080/02791072.2000.10400232
49. McKetin, R., Najman, J. M., Baker, A. L., Lubman, D. I., Dawe, S., Ali, R., . . . Mamun, A. (2012). Evaluating the impact of community-based treatment options on methamphetamine use: findings from the Methamphetamine Treatment Evaluation Study (MATES). *Addiction (Abingdon, England), 107*(11), 1998-2008. doi:10.1111/j.1360-0443.2012.03933.x
50. MCKETIN, R., DUNLOP, A. J., HOLLAND, R. M., SUTHERLAND, R. A., BAKER, A. L., SALMON, A. M., & HUDSON, S. L. (2013). Treatment outcomes for methamphetamine users receiving outpatient counselling from the Stimulant Treatment Program in Australia. *32*(1), 80-87. doi:<https://doi.org/10.1111/j.1465-3362.2012.00471.x>
51. Radfar, S. R., Mohsenifar, S., & Noroozi, A. (2017). Integration of Methamphetamine Harm Reduction into Opioid Harm Reduction Services in Iran: Preliminary Results of a Pilot Study. *11*(2), e7730. doi:10.5812/ijpbs.7730
52. **Mindfulness**
53. Carrico, A. W., Gómez, W., Siever, M. D., Discepola, M. V., Dilworth, S. E., & Moskowitz, J. T. (2015). Pilot randomized controlled trial of an integrative intervention with methamphetamine-using men who have sex with men. *Arch Sex Behav, 44*(7), 1861-1867. doi:10.1007/s10508-015-0505-5
54. Carrico, A. W., Nation, A., Gómez, W., Sundberg, J., Dilworth, S. E., Johnson, M. O., . . . Rose, C. D. (2015). Pilot trial of an expressive writing intervention with HIV-positive methamphetamine-using men who have sex with men. *Psychol Addict Behav, 29*(2), 277-282. doi:10.1037/adb0000031
55. **12 Steps facilitation**
56. Donovan, D. M. P. D., Daley, D. C. P. D., Brigham, G. S. P. D., Hodgkins, C. C. P. D., Perl, H. I. P. D., Garrett, S. B. M. P. H., . . . Zammarelli, L. M. A. (2013). Stimulant abuser groups to engage in 12-Step: A multisite trial in the National Institute on Drug Abuse Clinical Trials Network. *Journal of substance abuse treatment, 44*(1), 103-114. doi:10.1016/j.jsat.2012.04.004
57. **Family therapy**
58. Ghasemi, A., Rahimi Foroshani, A., Kheibar, N., Latifi, M., Khanjani, N., Eshagh Afkari, M., . . . Dastoorpour, M. (2014). Effects of family-centered empowerment model based education program on quality of life in methamphetamine users and their families. *Iranian Red Crescent medical journal, 16*(3), e13375-e13375. doi:10.5812/ircmj.13375
59. Wu, E., El-Bassel, N., Donald McVinney, L., Hess, L., Remien, R. H., Charania, M., & Mansergh, G. (2011). Feasibility and Promise of a Couple-Based HIV/STI Preventive Intervention for Methamphetamine-Using, Black Men Who have Sex with Men. *AIDS and Behavior, 15*(8), 1745-1754. doi:10.1007/s10461-011-9997-8
