# Supplementary material for: Psychosocial Interventions for Amphetamine Type Stimulant Use Disorder: An Overview of Systematic Reviews
Source: Front Psychiatry. 2021 Jun 17;12:512076. doi: 10.3389/fpsyt.2021.512076 (PMC8245759; doi:10.3389/fpsyt.2021.512076)
Supplement: Supplementary file 6 [file Table_6.pdf]

Author(s): Mai Thi Ngoc Tran, Quang Hung Luong, Giang Le Minh, Michael Dunne, Philip Baker

Question: Psycho-social intervention compared to Control for drug misuse

Certainty assessment							№ of patients		Effect		Certainty	Importance
№ of studies	Study design	Risk of bias	Inconsistency	Indirectness	Imprecision	Other considerations	Psycho-social intervention	Control	Relative (95% CI)	Absolute (95% CI)		
**% Drug use at the end of treatment**												
18	randomised trials	not serious	not serious	not serious	not serious	strong association all plausible residual confounding would reduce the demonstrated effect	663/1571 (42.2%)	888/1613 (55.1%)	**RR 0.80** (0.75 to 0.85)	**110 fewer per 1,000** (from 138 fewer to 83 fewer)	⊕⊕⊕⊕ HIGH	CRITICAL [b]
**Number of days using drugs in the past 30 days**												
9	randomised trials	not serious	not serious	not serious	not serious	none	413	742	-	MD **0 -1.59** (2.36 lower to 0.67 lower)	⊕⊕⊕⊕ HIGH [a]	CRITICAL [a]
**% Follow up treatment until the end of intervention with Contingency Management compared to Non-contingency management**												
7	randomised trials	not serious	not serious	not serious	not serious	strong association	289/519 (55.7%)	207/527 (39.3%)	**RR 1.42** (1.25 to 1.62)	**165 more per 1,000** (from 98 more to 244 more)	⊕⊕⊕⊕ HIGH	CRITICAL [d]
**% Follow up treatment until the end of intervention with CBT/MI compared to Treat as usual**												
4	randomised trials	not serious	not serious	not serious	not serious	none	341/558 (61.1%)	383/557 (68.8%)	**RR 0.89** (0.82 to 0.97)	**76 fewer per 1,000** (from 124 fewer to 21 fewer)	⊕⊕⊕⊕ HIGH	CRITICAL [d]
**% report unsafe sex at the end of CBT treatment compared to the control group**												
2	randomised trials	Serious [e]	not serious	not serious	not serious	none	50/249 (20.1%)	206/535 (38.5%)	**RR 0.49** (0.34 to 0.71)	**196 fewer per 1,000** (from 254 fewer to 112 fewer)	⊕⊕⊕◯ MODERATE	IMPORTANT [b]
**% Injection drug at the end of Psychosocial treatment compared to control**												

Certainty assessment							№ of patients		Effect		Certainty	Importance
№ of studies	Study design	Risk of bias	Inconsistency	Indirectness	Imprecision	Other considerations	Psycho-social intervention	Control	Relative (95% CI)	Absolute (95% CI)		
2	observational studies [f]	not serious	not serious	not serious	not serious	none	46/793 (5.8%)	126/807 (15.6%)	**OR 0.35** (0.24 to 0.49)	**95 fewer per 1,000** (from 114 fewer to 73 fewer)	⨁⨁◯◯ LOW	IMPORTANT [b]
**Beck Depression Inventory score at the end of Psychosocial treatment compared to control**												
5	observational studies	not serious	not serious	not serious	not serious	none	441	538	-	MD **0 -9.51** (10.67 lower to 8.35 lower)	⨁⨁⨁⨁ HIGH	CRITICAL [c]

CI: Confidence interval; **RR:** Risk ratio; **MD:** Mean difference; **OR:** Odds ratio

Explanations

a. A combination of different therapies (Eg: CBT+CM) could reduce 1.5 days of drug use compared to Cognitive behaviour therapy only. CBT can reduce 3 days of drug use per month compared to alternative treatment. The quality of evidence is moderate because some RCT showed a higher mean difference between intervention and control group
b. % drug use; % inject drugs, % unsafe sex behaviours at the end of people who use ATS in the psychosocial treatment group is significantly lower than in the control group
c. Beck Depression Inventory score at the end is considerably lower than at the beginning of psychosocial treatment.
d. However, Cognitive behaviour interventions are difficult in retaining participants in the treatment. Combination with Contingency Management can improve the retention rate.
e. There are two primary studies included in analysis. Strona (2006) had a high risk of bias because of small sample size
f. Although the included primary studies are Randomized control trials, the outcome was reported as an observation before and after the intervention.
