# Supplementary material for: Psychosocial Interventions for Amphetamine Type Stimulant Use Disorder: An Overview of Systematic Reviews
Source: Front Psychiatry. 2021 Jun 17;12:512076. doi: 10.3389/fpsyt.2021.512076 (PMC8245759; doi:10.3389/fpsyt.2021.512076)
Supplement: Supplementary file 7 [file Table_7.pdf]

## SUPPLEMENT 1: SEARCH STRATEGY

### Key concepts searches

| Topic | Key terms |
|---|---|
| **Amphetamine Type Stimulants** | *Amphetamine, *Methamphetamine, Meth, Ecstasy, MDMA, Psychostimulants |
| **Drug users** | Drug/Substance abuse*<br>Depend*<br>Disorder*<br>Addiction<br>Addicted<br>Illicit drug<br>Misuse |
| **Systematic Reviews** | Reviews<br>Meta-analysis |
| **Psychosocial** | Psychological/ Mindfulness/ Behaviour /Case management/ Contingency management Motivational interviewing/ Twelve steps/ Matrix/ Family/ Computer-based/ Internet-based |

### List of search statement technique

| | |
|---|---|
| **Statement 1** | (“*Amphetamine” OR “ *Methamphetamine” OR “Ecstasy” OR “Meth” OR “Psycho-stimulants” OR “MDMA”) **AND** (“Review*” OR “Systematic review*” OR “Meta-analysis”) |
| **Statement 2** | “Drug users” OR “Illicit drug” OR “Drug abuse*” OR “Addict*” OR “Misuse” OR “Depend*” OR “Drug Disorder*” **AND** (“Review*” OR “Systematic review*”OR “Meta-analysis”) |

**Results on 28 December 2020**

| No | Search query | Search result | | | | | | | | |
|---|---|---|---|---|---|---|---|---|---|---|
| | | **Cochrane** | **Health Evidence** | **DARE** | **Prospero** | **Medline Via Ebscohost** | **Pubmed** | **Scopus** | **Web of Science** | **Sum** |
| # 1 | Statement 1 | 51 | 22 | 18 | 160 | 23 | 84 | 46 | 82 | |
| # 2 | Statement 2 | 186 | 542 | 48 | 423 | 550 | 183 | 328 | 2335 | |
| # 3 | #2 OR #1 | | | | | | | | | |
| # 4 | #3 AND excluding document types | | | | | | | | | |
| | Refined by: [excluding] Document Types: ( Meeting Abstract Or Proceedings Paper Or Editorial Material Or Book Chapter Or Book Review Or Retracted Publication Or Biographical Item Or Correction Addition Or Discussion Or Data Paper Or Retraction Or Bibliography Or Software Review Or Tv Review Or Radio Review ) | | | | | | | | | |
| # 5 | #4 AND excluding languages and other publications | 182 | 532 | 45 | 184 | 328 | 198 | 225 | 195 | |
| | Refined by: [excluding] Types: ( Meeting Abstract Or Proceedings Paper Or Editorial Material Or Book Chapter Or Letter Or Correction Or Note Or News Item Or Book Or Reprint Or Early Access Or Retracted Publication Or Biographical Item Or Correction Addition Or Discussion Or Data Paper Or Bibliography Or Book Review Or Software Review Or TV Review Or Radio Review) AND AND [excluding]Languages: ( German Or Spanish Or French Or Portuguese Or Russian Or Turkish Or Polish Or Italian Or Korean Or Czech Or Hungarian Or Croatian Or Greek Or Dutch Or Japanese Or Slovenian Or Slovak Or Lithuanian Or Serbian Or | | | | | | | | | |

| | | | | | | | | | | |
|---|---|---|---|---|---|---|---|---|---|---|
| | Persian Or Malay Or Romanian Or Chinese Or Icelandic Or Arabic Or Afrikaans Or Norwegian Or Ukrainian Or Danish Or Catalan Or Swedish Or Estonian Or Bulgarian Or Serbo Croatian Or Galician Or Georgian Or Esperanto Or Finnish Or Hebrew Or Indonesian Or Welsh ) | | | | | | | | | |
| # 6 | #5 AND (Any Psychosocial intervention) | 78 | 198 | 17 | 28 | 93 | 58 | 72 | 57 | |
| # 7 | AU=("Anonymous" OR "anonymous") | | | | | | | | | |
| # 8 | #6 NOT #7 | 78 | 194 | 17 | 28 | 90 | 57 | 72 | 51 | 587 |

**Search by hand from included systematic reviews and websites (**The United Nations Office on Drugs and Crime UNODC, National Institute on Drug Abuse (NIDA), National Drug & Alcohol Research Centre (NDARC), NHMRC Centre of Research Excellence in Mental Health and Substance use): **8 systematic reviews.**
