# Supplementary material for: Psychosocial Interventions for Amphetamine Type Stimulant Use Disorder: An Overview of Systematic Reviews
Source: Front Psychiatry. 2021 Jun 17;12:512076. doi: 10.3389/fpsyt.2021.512076 (PMC8245759; doi:10.3389/fpsyt.2021.512076)
Supplement: Supplementary file 8 [file Table_8.DOCX]

**EXCLUDED SYSTEMATIC REVIEWS**

| **STT** | **SYSTEMATIC REVIEWS** | **REASON FOR EXCLUSION** |
| --- | --- | --- |
| 1 | Gibson, D. R., McCusker, J., & Chesney, M. (1998). Effectiveness of psychosocial interventions in preventing HIV risk behaviour in injecting drug users. *Aids, 12*(8), 919-929. | Did not include any primary studies among people who use ATS |
| 2 | Dunn, C., Deroo, L., & Rivara, F. P. (2001). The use of brief interventions adapted from motivational interviewing across behavioral domains: a systematic review. *Addiction, 96*(12), 1725-1742. doi:10.1080/09652140120089481 | Low quality |
| 3 | Prendergast, M. L., Urada, D., & Podus, D. (2001). Meta-analysis of HIV risk-reduction interventions within drug abuse treatment programs. *Journal of Consulting and Clinical Psychology, 69*(3), 389. doi:10.1037/0022-006X.69.3.389 | Did not include any primary studies among people who use ATS |
| 4 | van Empelen, P., Kok, G., van Kesteren, N. M., van den Borne, B., Bos, A. E., & Schaalma, H. P. (2003). Effective methods to change sex-risk among drug users: a review of psychosocial interventions. *Soc Sci Med, 57*(9), 1593-1608. | Did not include any primary studies among people who use ATS |
| 5 | Drake, R. E., Mueser, K. T., Brunette, M. F., & McHugo, G. J. (2004). A review of treatments for people with severe mental illnesses and co-occurring substance use disorders. *Psychiatr Rehabil J, 27*(4), 360-374. | Did not include any primary studies among people who use ATS |
| 6 | Roozen, H. G., Boulogne, J. J., van Tulder, M. W., van den Brink, W., De Jong, C. A., & Kerkhof, A. J. (2004). A systematic review of the effectiveness of the community reinforcement approach in alcohol, cocaine and opioid addiction. *Drug Alcohol Depend, 74*(1), 1-13. doi:10.1016/j.drugalcdep.2003.12.006 | Did not include any primary studies among people who use ATS |
| 7 | Mueser, K. T., Drake, R. E., Sigmon, S. C., & Brunette, M. F. (2005). Psychosocial Interventions for Adults with Severe Mental Illnesses and Co-Occurring Substance Use Disorders *Journal of Dual Diagnosis, 1*(2), 57-82. doi:10.1300/J374v01n02_05 | Did not include any primary studies among people who use ATS |
| 8 | Smith, L. A., Gates, S., & Foxcroft, D. (2006). Therapeutic communities for substance related disorder *Cochrane Database of Systematic Reviews*(1). doi:10.1002/14651858.CD005338.pub2 | Included one study among people who use ATS, however, did not separate outcomes among people who use ATS |
| 9 | Grenard, J. L., Ames, S. L., Pentz, M. A., & Sussman, S. (2006). Motivational interviewing with adolescents and young adults for drug-related problems. *Int J Adolesc Med Health, 18*(1), 53-67. | Did not include any primary studies among people who use ATS |
| 10 | Copenhaver, M. M., Johnson, B. T., Lee, I. C., Harman, J. J., & Carey, M. P. (2006). Behavioral HIV risk reduction among people who inject drugs: meta-analytic evidence of efficacy. *J Subst Abuse Treat, 31*(2), 163-171. doi:10.1016/j.jsat.2006.04.002 | Did not include any primary studies among people who use ATS |
| 11 | Hesse, M., Vanderplasschen, W., Rapp, R. C., Broekaert, E., & Fridell, M. (2007). Case management for persons with substance use disorders. *Cochrane Database Syst Rev*(4), Cd006265. doi:10.1002/14651858.CD006265.pub2 | There was one study, which targeted people who use ATS. However, outcome measurement was not separated for people who use ATS. |
| 12 | Dutra, L., Stathopoulou, G., Basden, S. L., Leyro, T. M., Powers, M. B., & Otto, M. W. (2008). A meta-analytic review of psychosocial interventions for substance use disorders. *Am J Psychiatry, 165*(2), 179-187. doi:10.1176/appi.ajp.2007.06111851 | Did not include any primary studies among people who use ATS |
| 13 | Drake, R. E., O'Neal, E. L., & Wallach, M. A. (2008). A systematic review of psychosocial research on psychosocial interventions for people with co-occurring severe mental and substance use disorders. *J Subst Abuse Treat, 34*(1), 123-138. doi:10.1016/j.jsat.2007.01.011 | Did not include any primary studies among people who use ATS |
| 14 | Zgierska, A., Rabago, D., Chawla, N., Kushner, K., Koehler, R., & Marlatt, A. (2009). Mindfulness meditation for substance use disorders: a systematic review *Subst Abus, 30*(4), 266-294. doi:10.1080/08897070903250019 | Did not include any primary studies among people who use ATS |
| 15 | Konghom, S., Verachai, V., Srisurapanont, M., Suwanmajo, S., Ranuwattananon, A., Kimsongneun, N., & Uttawichai, K. (2010). Treatment for inhalant dependence and abuse *Cochrane Database of Systematic Reviews*(12). doi:10.1002/14651858.CD007537.pub2 | Did not include any primary studies among people who use ATS |
| 16 | Meader, N., Li, R., Des Jarlais, D. C., & Pilling, S. (2010). Psychosocial interventions for reducing injection and sexual risk behaviour for preventing HIV in drug users *Cochrane Database Syst Rev*(1), Cd007192. doi:10.1002/14651858.CD007192.pub2 | Did not include any primary studies among people who use ATS |
| 17 | Smedslund, G., Berg, R. C., Hammerstrøm, K. T., Steiro, A., Leiknes, K. A., Dahl, H. M., & Karlsen, K. (2011). Motivational interviewing for substance abuse *Cochrane Database of Systematic Reviews*(5). doi:10.1002/14651858.CD008063.pub2 | Did not include any primary studies among people who use ATS |
| 18 | Amato, L., Davoli, M., Vecchi, S., Ali, R., Farrell, M., Faggiano, F., . . . Chengzheng, Z. (2011). Cochrane systematic reviews in the field of addiction: What's there and what should be. *Drug and Alcohol Dependence, 113*(2), 96-103. doi:<https://doi.org/10.1016/j.drugalcdep.2010.08.003> | Did not include any primary studies among people who use ATS |
| 19 | Hofmann, S. G., Asnaani, A., Vonk, I. J. J., Sawyer, A. T., & Fang, A. (2012). The Efficacy of Cognitive Behavioral Therapy: A Review of Meta-analyses. *Cognitive Therapy and Research, 36*(5), 427-440. doi:10.1007/s10608-012-9476-1 | High risk of bias – Low-quality evidence |
| 20 | Mbuagbaw, L., Ye, C., & Thabane, L. (2012). Motivational interviewing for improving outcomes in youth living with HIV. *Cochrane Database of Systematic Reviews*(9). doi:10.1002/14651858.CD009748.pub2 | Did not include any primary studies among people who use ATS |
| 21 | Naar-King, S., Parsons, J. T., & Johnson, A. M. (2012). Motivational Interviewing Targeting Risk Reduction for People with HIV: A Systematic Review. *Curr HIV/AIDS Rep, 9*(4), 335-343. doi:10.1007/s11904-012-0132-x | Did not include any primary studies among people who use ATS |
| 22 | van Dam, D., Vedel, E., Ehring, T., & Emmelkamp, P. M. (2012). Psychological treatments for concurrent posttraumatic stress disorder and substance use disorder: a systematic review *Clin Psychol Rev, 32*(3), 202-214. doi:10.1016/j.cpr.2012.01.004 | The low quality and did not include any primary studies among people who use ATS |
| 23 | Wechsberg, W., Golin, C., El-Bassel, N., Hopkins, J., & Zule, W. (2012). Current Interventions to Reduce Sexual Risk Behaviors and Crack Cocaine Use among HIV-Infected Individuals. *Curr HIV/AIDS Rep, 9*(4), 385-393. doi:10.1007/s11904-012-0131-y | Did not include any primary studies among people who use ATS |
| 24 | Rajasingham, R., Mimiaga, M. J., White, J. M., Pinkston, M. M., Baden, R. P., & Mitty, J. A. (2012). A Systematic Review of Behavioral and Treatment Outcome Studies Among HIV-Infected Men Who Have Sex with Men Who Abuse Crystal Methamphetamine. *AIDS Patient Care and STDs, 26*(1), 36-52. doi:10.1089/apc.2011.0153 | Did not include any primary studies among people who use ATS |
| 25 | Hunt, G. E., Siegfried, N., Morley, K., Sitharthan, T., & Cleary, M. (2013). Psychosocial interventions for people with both severe mental illness and substance misuse. *Cochrane Database of Systematic Reviews*(10). doi:10.1002/14651858.CD001088.pub3 | 3 in 32 included studies are among people who use ATS. However, outcome measurement was not separated for people who use ATS. |
| 26 | Werb, D., Buxton, J., Shoveller, J., Richardson, C., Rowell, G., & Wood, E. (2013). Interventions to prevent the initiation of injection drug use: A systematic review. *Drug and Alcohol Dependence, 133*(2), 669-676. doi:<https://doi.org/10.1016/j.drugalcdep.2013.08.017> | Did not include any primary studies among people who use ATS |
| 27 | Wang, N., Sun, X., Yin, L., Liu, H., Ruan, Y., Shao, Y., . . . Vermund, S. H. (2013). Meta-Analysis of Interventions for Reducing Number of Sexual Partners and Drug and Alcohol Abuse among People Living with HIV/AIDS. *Journal of AIDS & clinical research, 4*, 14272. doi:10.4172/2155-6113.1000213 | Did not include any primary studies among people who use ATS |
| 28 | Chiesa, A., & Serretti, A. (2014). Are Mindfulness-Based Interventions Effective for Substance Use Disorders? A Systematic Review of the Evidence *Substance use & misuse, 49*(5), 492-512. doi:10.3109/10826084.2013.770027 | Including one primary study among people who use ATS but it existed in other reviews. Low quality of evidence |
| 29 | Wood, S. K., Eckley, L., Hughes, K., Hardcastle, K. A., Bellis, M. A., Schrooten, J., . . . Voorham, L. (2014). Computer-based programmes for the prevention and management of illicit recreational drug use: A systematic review *Addictive behaviors, 39*(1), 30-38. doi:10.1016/j.addbeh.2013.09.010 | The low quality and did not include any primary studies among people who use ATS. Participants are both students and drug users |
| 30 | Klimas, J., Tobin, H., Field, C. A., O'Gorman, C. S., Glynn, L. G., Keenan, E., . . . Cullen, W. (2014). Psychosocial interventions to reduce alcohol consumption in concurrent problem alcohol and illicit drug users. *Cochrane Database Syst Rev*(12), Cd009269. doi:10.1002/14651858.CD009269.pub3 | Did not include any primary studies among people who use ATS |
| 31 | Knapp, W. P., Soares, B. G. O., Farrell, M. F., & Silva de Lima, M. (2015). Psychosocial interventions for cocaine and psychostimulant amphetamines related disorders *Cochrane Database of Systematic Reviews*(4). doi:10.1002/14651858.CD003023.pub3 | Review withdrawn as the Cochrane Funding Arbitration Panel found this review non-compliant with the Cochrane’s commercial sponsorship policy. The new author team has updated in 2016 and replaced this review. |
| 32 | Melendez-Torres, G. J., & Bonell, C. (2014). Systematic review of cognitive behavioural interventions for HIV risk reduction in substance-using men who have sex with men. *Int J STD AIDS, 25*(9), 627-635. doi:10.1177/0956462413515638 | Did not include any primary studies among people who use ATS |
| 33 | Srisurapanont, M., Jarusuraisin, N., Kittirattanapaiboon, P., & Kao, U. (2014). Treatment for amphetamine dependence and abuse. *Cochrane Database of Systematic Reviews*(4). doi:10.1002/14651858.CD003022.pub2 | Did not include any primary studies among people who use ATS |
| 34 | Allara, E., Ferri, M., Bo, A., Gasparrini, A., & Faggiano, F. (2015). Are mass-media campaigns effective in preventing drug use? A Cochrane systematic review and meta-analysis. *BMJ Open, 5*(9), e007449. doi:10.1136/bmjopen-2014-007449 | Included one study among people who use ATS.  Did not separate outcome among people who use ATS |
| 35 | Jeal, N., Macleod, J., Turner, K., & Salisbury, C. (2015). Systematic review of interventions to reduce illicit drug use in female drug-dependent street sex workers. *BMJ Open, 5*(11), e009238. doi:10.1136/bmjopen-2015-009238 | Did not include any primary studies among people who use ATS |
| 36 | Perry, A. E., Neilson, M., Martyn‐St James, M., Glanville, J. M., Woodhouse, R., & Hewitt, C. (2015). Interventions for female drug‐using offenders *Cochrane Database of Systematic Reviews*(6). doi:10.1002/14651858.CD010910.pub2 | Did not include any primary studies among people who use ATS |
| 37 | Terplan, M., Ramanadhan, S., Locke, A., Longinaker, N., & Lui, S. (2015). Psychosocial interventions for pregnant women in outpatient illicit drug treatment programs compared to other interventions *Cochrane Database of Systematic Reviews*(4). doi:10.1002/14651858.CD006037.pub3 | Did not include any primary studies among people who use ATS |
| 38 | Cumming, C., Troeung, L., Young, J. T., Kelty, E., & Preen, D. B. (2016). Barriers to accessing methamphetamine treatment: A systematic review and meta-analysis. *Drug and Alcohol Dependence, 168*, 263-273. doi:<https://doi.org/10.1016/j.drugalcdep.2016.10.001> | Did not include any primary studies among people who use ATS |
| 39 | Joo, J. Y., & Huber, D. L. (2015). Community-based case management effectiveness in populations that abuse substances *Int Nurs Rev, 62*(4), 536-546. doi:10.1111/inr.12201 | Did not include any primary studies among people who use ATS |
| 40 | Roberts, N., Roberts, P. A., Jones, N., & Bisson, J. (2016). Psychological therapies for post-traumatic stress disorder and comorbid substance use disorder *Cochrane Database of Systematic Reviews, 2016*(4). doi:10.1002/14651858.CD010204.pub2 | Did not include any primary studies among people who use ATS |
| 41 | Davis, D. R., Kurti, A. N., Skelly, J. M., Redner, R., White, T. J., & Higgins, S. T. (2016). A review of the literature on contingency management in the treatment of substance use disorders, 2009-2014. *Prev Med, 92*, 36-46. doi:10.1016/j.ypmed.2016.08.008 | 2 among 69 included studies were among people who use ATS |
| 42 | Tang, Y.-Y., Tang, R., & Posner, M. I. (2016). Mindfulness meditation improves emotion regulation and reduces drug abuse. *Drug and Alcohol Dependence, 163*, S13-S18. doi:<https://doi.org/10.1016/j.drugalcdep.2015.11.041> | Did not include any primary studies among people who use ATS |
| 43 | Boumparis, N., Karyotaki, E., Schaub, M. P., Cuijpers, P., & Riper, H. (2017). Internet interventions for adult illicit substance users: a meta-analysis. *Addiction, 112*(9), 1521-1532. doi:10.1111/add.13819 | Included two studies among people who use ATS but it existed in other reviews.  Did not separate outcome among people who use ATS |
| 44 | Davis, J. P., Smith, D. C., & Briley, D. A. (2017). Substance use prevention and treatment outcomes for emerging adults in non-college settings: A meta-analysis. *Psychology of addictive behaviors : journal of the Society of Psychologists in Addictive Behaviors, 31*(3), 242. doi:10.1037/adb0000267 | Did not include any primary studies among people who use ATS |
| 45 | Jiang, S., Wu, L., & Gao, X. (2017). Beyond face-to-face individual counseling: A systematic review on alternative modes of motivational interviewing in substance abuse treatment and prevention. *Addict Behav, 73*, 216-235. doi:10.1016/j.addbeh.2017.05.023 | Not have a specific outcome for ATS use |
| 46 | Herrmann, E. S., Matusiewicz, A. K., Stitzer, M. L., Higgins, S. T., Sigmon, S. C., & Heil, S. H. (2017). Contingency Management Interventions for HIV, Tuberculosis, and Hepatitis Control Among Individuals With Substance Use Disorders: A Systematized Review. *Journal of substance abuse treatment, 72*, 117-125. doi:10.1016/j.jsat.2016.06.009 | Did not include any primary studies among people who use ATS |
| 47 | Li, W., Howard, M. O., Garland, E. L., McGovern, P., & Lazar, M. (2017). Mindfulness treatment for substance misuse: A systematic review and meta-analysis. *J Subst Abuse Treat, 75*, 62-96. doi:10.1016/j.jsat.2017.01.008 | Did not include any primary studies among people who use ATS |
| 48 | Weissman, J., Kanamori, M., Devieux, J. G., Trepka, M. J., & De La Rosa, M. (2017). HIV Risk Reduction Interventions Among substance-abusing Reproductive-Age Women: A Systematic Review *AIDS Educ Prev, 29*(2), 121-140. doi:10.1521/aeap.2017.29.2.121 | Included one study among people who use ATS.  Did not separate outcome among people who use ATS |
| 49 | Martinez-Vispo, C., Martinez, U., Lopez-Duran, A., Fernandez Del Rio, E., & Becona, E. (2018). Effects of behavioural activation on substance use and depression: a systematic review *Subst Abuse Treat Prev Policy, 13*(1), 36. doi:10.1186/s13011-018-0173-2 | Including four studies among people who use ATS, but they existed in other reviews |
| 50 | Moreland, A. D., & McRae-Clark, A. (2018). Parenting outcomes of parenting interventions in integrated substance-use treatment programs: A systematic review. *Journal of substance abuse treatment, 89*, 52-59. doi:10.1016/j.jsat.2018.03.005 | Did not include any primary studies among people who use ATS |
| 51 | Pennington, B., Collins, B., Leigh, S., Martin, A. P., Owen, L., Fischer, A., . . . Bates, G. (2018). The cost-effectiveness of seven behavioural interventions to prevent drug misuse in vulnerable populations. *International Journal of Drug Policy, 57*, 42-50. doi:<https://doi.org/10.1016/j.drugpo.2018.03.028> | Different outcome measurement. They measured cost-effectiveness. |
| 52 | Coco, G. L., Melchiori, F., Oieni, V., Infurna, M. R., Strauss, B., Schwartze, D., . . . Gullo, S. (2019). Group treatment for substance use disorder in adults: A systematic review and meta-analysis of randomized-controlled trials. *Journal of substance abuse treatment*. doi:10.1016/j.jsat.2019.01.016 | Did not include any primary studies among people who use ATS |
| 53 | Liu, F., Cui, J., Liu, X., Chen, K. W., Chen, X., Li, R. J. B. C. M., & Therapies. (2020). The effect of tai chi and Qigong exercise on depression and anxiety of individuals with substance use disorders: a systematic review and meta-analysis. *20*, 1-11. | Include only one RCT among ATS users about the effect of Taichi on depression among female ATS users  But the intervention is not focussing on psychosocial intervention |
| 54 | Lappan, S. N., Brown, A. W., & Hendricks, P. S. (2020). Dropout rates of in-person psychosocial substance use disorder treatments: a systematic review and meta-analysis. *Addiction, 115*(2), 201-217. doi:10.1111/add.14793 | Did not include any primary studies among people who use ATS |
| 55 | Green, B., Hayllar, J., & Anderson, C. (2020). Speeding: a review of self-reported effects of amphetamine-type stimulants (ATS). *Addiction Research & Theory, 28*(1), 82-90. doi:10.1080/16066359.2019.1622000 | Out of scope: No intervention |
| 56 | Horrell, J., Thompson, T. P., Taylor, A. H., Neale, J., Husk, K., Wanner, A., . . . Wallace, G. (2020). Qualitative systematic review of the acceptability, feasibility, barriers, facilitators and perceived utility of using physical activity in the reduction of and abstinence from alcohol and other drug use. *Mental Health and Physical Activity, 19*, 100355. doi:<https://doi.org/10.1016/j.mhpa.2020.100355> | Physical activities intervention |
| 57 | Heijdra Suasnabar, J. M., & Hipple Walters, B. (2020). Community-based psychosocial substance use disorder interventions in low-and-middle-income countries: a narrative literature review. *International Journal of Mental Health Systems, 14*(1), 74. doi:10.1186/s13033-020-00405-3 | Only one study about methamphetamine |
